# Determination of the characteristic curves of a nonlinear first order system from Fourier analysis

**DOI:** 10.1038/s41598-023-29151-5

**Published:** 2023-02-02

**Authors:** Federico J. Gonzalez

**Affiliations:** 1 Instituto de Física Rosario (CONICET-UNR), Bv. 27 de Febrero 210 Bis, Rosario, S2000EZP Argentina; 2 Facultad de Ciencias Exactas, Ingeniería y Agrimensura (UNR), Av. Pellegrini 250, Rosario, S2000BTP Argentina

**Keywords:** Electrical and electronic engineering, Mechanical engineering, Applied mathematics, Nonlinear phenomena

## Abstract

Based on Fourier analysis, we develop an expression for modeling and simulating nonlinear first order systems. This expression is associated to a nonlinear first order differential equation $y=f(x)+g(x)x'$, where $x=x(t)$ is the dynamical variable, $y=y(t)$ is the driving force, and the *f* and *g* functions are the characteristic curves which are associated to dissipative and memory elements, respectively. The model is obtained from the sinusoidal response, specifically by calculating the Fourier analysis of *y*(*t*) for $x(t)=A_1\sin (\omega t)+A_0$, where the model parameters are the Fourier coefficients of the response, and the values of $A_0$, $A_1$ and $A_1'=A_1\omega$. The same expression is used for two kinds of time-domain simulations: to calculate other driving force $\hat{y}$ based on a dynamical variable $\hat{x}$; and, to calculate the dynamical variable $\hat{x}$ based on a driving force $\hat{y}$. In both cases, the dynamical variable must remain in the range $\hat{x}\in [A_0-A_1,A_0+A_1]$. By analyzing this expression, we found an equivalence between the Fourier coefficients and the polynomial regressions of the characteristic curves of *f* and *g*. This result allows us to obtain the system modeling and simulation based on the amplitude and phase Fourier spectrum obtained from the Fast Fourier Transform (FFT) of the sampled $y_n$ version of *y*(*t*). It is shown that this technique has a low computational complexity, and it is expected to be suitable for real-time applications for system modeling, simulation and control, in particular when the explicit expressions of the characteristic curves are unknown. Fourier analysis is a fundamental tool in electronics, mathematics and physics, but to the best of the author’s knowledge, no work has found this clear evidence of the connection between the Fourier analysis and a first order differential equation. The aim of this work is to initiate a systematic study on this topic.

## Introduction

Nonlinear dynamical systems have been extensively studied in the last century, and as a result a wide variety of methods have been developed. Introductory books and modern reviews can be found for: circuit analysis and control systems in Refs.^1–7^; structural dynamics and vibrations in Refs.^8–12^; electrochemistry in Refs.^13–17^, general physics and applied mathematics in Refs.^18–24^. Probably, the earlier studies of nonlinear systems are the Volterra series^25,26^ in the 1890s and the Wiener series^27,28^ in the 1940s and 1950s. Simultaneously, the contributions of Poincaré^29^ in the 1890s, Van der Pol^30^, Lienard^31^ and Fatou^32^ in the 1920s, and Bogolyubov, Krylov and Mitropolskii^33–36^ in the 1930s and 1940s about nonlinear systems have led to the standard theories of the averaging and perturbation methods, Harmonic Balance Method (HBM) and Describing Functions (DFs)^37^. The practical implementation of these methods has led to the developments of the *n*onlinear *a*uto*r*egressive *m*oving *a*verage model with e*x*ogenous inputs (NARMAX model)^38,39^ in the 1980s, and the Wiener model, the Hammerstein model and the Wiener–Hammerstein model in the 2000s (see Ref.^40^ and references therein).

Fourier analysis is a powerful tool based on harmonic analysis that allows us to study a time-domain problem from its frequency-domain representation. Linear differential equations can be studied by unified frameworks such as the transfer function (TF) in electrical and electronic engineering, the frequency response function (FRF) in mechanical engineering and the Green’s function in physics. These frameworks allow us to calculate all possible linear behaviors in the frequency-domain by using the first harmonic. However, nonlinear differential equations present higher harmonics that can not be avoided. Many methods have been developed in order to include higher harmonics such as: the Generalized Frequency Response Functions (GFRFs)^41^ in the 1950s; the Incremental Harmonic Balance (IHB)^42–45^, the Alternating Frequency/Time Domain Method (AFT)^46^ and the Higher-Order Frequency Response Functions (HOFRFs)^47^ in the 1980s; the Nonlinear Output Frequency Response Function (NOFRF)^48^, the Output Frequency Response Function (OFRF)^49–51^, the Higher-Order Sinusoidal Input Describing Functions (HOSIDFs)^52^ and many modified versions of HBM and IHB^53–59^ in the 1990s, 2000s and 2010s.

Modern reviews of these methods can be found in Refs.^60–64^. According to Ref.^60^, an ultimate goal of the frequency-domain analysis is to “provide a clear explicit relationship for the system output spectrum and the nonlinear characteristic parameters”. In this direction, the OFRF method has provided us an explicit relationship, however, a recursive algorithm is required in order to obtain these coefficients^51^. In addition, the HOSIDFs method introduces a “virtual harmonic generator” which may unintentionally disguise the relation between the Fourier spectrum and the physical parameters of the nonlinear system.

This work is a contribution in order to define a unified framework to relate all the higher harmonics with the physical parameters of the nonlinear system, avoiding recursive algorithms and virtual generators. We are interested in addressing these questions: how do we obtain as much information as possible about the nonlinear system from the harmonics?; and more specifically, what is the relationship between the harmonics in the system and the analytical expressions which define equations of the nonlinear system?

To address these questions we present a formalism which is based on Fourier analysis from the perspective of the electrical and electronics engineering^65–68^, but aiming also to be applied into other areas as a multidisciplinary tool. The word harmonics in this context refers to the Fourier spectrum obtained from the Fast Fourier Transform (FFT) of a sampled continuous variable. This formalism is motivated from the existent bibliography, mainly by the OFRF and HOSIDFs methods, but, to the best of the author’s knowledge, it is conceptually new. We have considered a reformulation of the Fourier series in terms of a power series expression, and as a result, we found analytical expressions which show clear evidence of the relation between the Fourier analysis and the characteristic curves of a first order system. These analytical expressions can be applied to system modeling and simulating from input–output data without using recursive algorithms or virtual generators. This formalism implies that by calculating the Fourier spectrum of the driving force with a fixed sinusoidal dynamical variable, we are able to obtain a complete description of the characteristic curves of the nonlinear system. Furthermore, as it is shown in this work, these expressions show an equivalence between the Fourier analysis and two polynomial fittings based on polynomial expansions for the characteristic curves. In summary, this work aims to initiate a systematic study on the topic of determination of the nonlinear characteristic curves from Fourier analysis, and the starting point to be addressed here is the first order system.

Technically, the expressions of this work are based on rewriting the Fourier series for the driving force *y*(*t*) into a convenient power series expression of the dynamical variable *x*(*t*). These expressions has a functional dependence which can be identified as a first order system $y(t)= f(x(t))+g(x(t))x'(t)$. By using these expressions, the model of the system, which is defined by the *f* and *g* functions so-called characteristic curves, can be obtained from a sinusoidal dynamical variable $x(t)=A_1 \sin (\omega t)+A_0$, where the model parameters are the Fourier coefficients of the driving force, and the values of $A_0$, $A_1$ and $A'_1=\omega A_1$. The Fourier analysis of the driving force up to a given order $k_{max}$ allows us to obtain the characteristic curves, within the range $x\in [A_0-A_1,A_0+A_1]$, where the characteristic curves *f* and *g* are defined by a polynomial expansion up to order $k_{max}$ and $k_{max}-1$, respectively. We demonstrate that this equivalence between the Fourier analysis of the driving force and the polynomial expansions of the characteristic curves is in fact an equivalence between the Fourier analysis of the driving force and the polynomial curve fittings of the characteristic curves. Due to this equivalence, and by noticing that the dynamical variable is the input in the modeling stage, the method is proposed to be called: “Sinusoidal Input Response in Power Series (SIRPS)”.

Once the system is estimated from the Fourier analysis of the sinusoidal response, we obtain the characteristic curves that can be used for a time-domain simulation in two ways: (1) by calculating the driving force that corresponds to a giving dynamical variable; and (2) by calculating the dynamical variable that corresponds to a giving driving force. Finally, the method can be considered as a combined time-frequency approach, because the system estimation is based on the frequency-domain method and the simulation is based on the time-domain method.

## Results

### Formalism

We are interested in nonlinear dynamical systems that can be modeled by the first order equation

1 $$\begin{aligned} y(t)= f(x(t))+g(x(t))\; x'(t) , \end{aligned}$$

where *x*(*t*) is the dynamical variable of the system and *y*(*t*) is a driving force, they will be referred to as the input and the response of the system, respectively. Furthermore, *f* and *g* are nonlinear functions of the dynamical variable. In circuit theory, the *f* and *g* functions are called characteristic curves, because they describe the system. If these curves are known, then Eq. (1) can be solved by using some numerical technique or direct integration. Notice that the left side has explicit dependence on the independent variable *t*, but the right side does not, since it depends on the dynamical variable and its first derivative. This observation is discussed in more detail in the *Applications* section to elucidate the range of nonlinear systems that can be represented by Eq. (1).

Suppose that *y*(*t*) is a periodic function with period *T* that satisfies the Dirichlet conditions^69^ for Fourier series existence, see Ref.^65^ for a modern exposition of the subject, then *y*(*t*) can be expressed as a Fourier series

2 $$\begin{aligned} y(t)=a_0+\sum _{k=1}^\infty ( a_k \cos (k\omega t) + b_k \sin (k\omega t) ) , \end{aligned}$$

where $\omega =2\pi /T$ is the fundamental frequency and the Fourier coefficients are calculated by

3 $$\begin{aligned} \begin{aligned} {\left\{ \begin{array}{ll} a_0 &{}= \frac{\omega }{\pi }\int _{0}^{2\pi /\omega } y(t) dt \\ a_k &{}= \frac{\omega }{2\pi }\int _{0}^{2\pi /\omega } y(t) \cos (k\omega t) dt , \,k\ge 1 \\ b_k &{}= \frac{\omega }{2\pi }\int _{0}^{2\pi /\omega } y(t) \sin (k\omega t) dt , k\ge 1 . \end{array}\right. } \end{aligned} \end{aligned}$$

By using the multiple-angle formulas

4 $$\begin{aligned} \sin (kx)&=\frac{e^{ikx}-e^{-ikx}}{2i}=\frac{(\cos (x)+i\sin (x))^k-(\cos (x)-i\sin (x))^k}{2i}\nonumber \\&=\sum _{l=0}^k \left( {\begin{array}{c}k\\ l\end{array}}\right) \cos ^l(x) \sin ^{k-l}(x) \sin \left( \frac{\pi }{2}(k-l) \right) \end{aligned}$$

and

5 $$\begin{aligned} \cos (kx)&=\frac{e^{ikx}+e^{-ikx}}{2} =\sum _{l=0}^k \left( {\begin{array}{c}k\\ l\end{array}}\right) \cos ^l(x) \sin ^{k-l}(x) \cos \left( \frac{\pi }{2}(k-l) \right) \end{aligned}$$

where the Euler formula and binomial theorem were used, and $$\left( {\begin{array}{c}k\\ l\end{array}}\right)$$ is the binomial coefficient, then we can rewrite Eq. (2) as

6 $$\begin{aligned} y(t)=a_0+\sum _{k=1}^\infty \sum _{l=0}^k \left( {\begin{array}{c}k\\ l\end{array}}\right) \cos ^l(\omega t) \sin ^{k-l}(\omega t) (ab)_{kl} , \end{aligned}$$

where

7 $$\begin{aligned} (ab)_{kl}{:=}a_k \cos \left( \frac{\pi }{2}(k-l)\right) +b_k \sin \left( \frac{\pi }{2}(k-l)\right) \end{aligned}$$

contains the Fourier coefficients $a_k$ and $b_k$. A further manipulation can be made by using Pythagorean identity $\sin ^2(x)+\cos ^2(x)=1$. We use the expressions

8 $$\begin{aligned} \cos ^l(\omega t)={\left\{ \begin{array}{ll} \left( 1-\sin ^2(\omega t)\right) ^{l/2} &{} \text {if l is even} \\ \cos ^{l-1}(\omega t)\cos (\omega t) &{} \text {if l is odd} \end{array}\right. } ={\left\{ \begin{array}{ll} \left( 1-\sin ^2(\omega t)\right) ^{l/2} &{} \text {if l is even} \\ \left( 1-\sin ^2(\omega t)\right) ^{(l-1)/2}\cos (\omega t) &{} \text {if l is odd} \end{array}\right. } \end{aligned}$$

to rewrite Eq. (6) into

9 $$\begin{aligned} \begin{aligned} y(t)&= \left[ a_0 + \sum _{k=1}^\infty \sum _{\begin{array}{c} l=0\\ l=l+2 \end{array}}^k \left( {\begin{array}{c}k\\ l\end{array}}\right) \left( 1-\sin ^2(\omega t)\right) ^{l/2}\sin ^{k-l}(\omega t) (ab)_{kl} \right] \\&\quad + \left[ \sum _{k=1}^\infty \sum _{\begin{array}{c} l=1\\ l=l+2 \end{array}}^k \left( {\begin{array}{c}k\\ l\end{array}}\right) \left( 1-\sin ^2(\omega t)\right) ^{(l-1)/2}\sin ^{k-l}(\omega t) (ab)_{kl} \right] \cos (\omega t) . \end{aligned} \end{aligned}$$

Note that, based on Eq. (6) and by using the expressions of Eq. (8), there is no other way to write Eq. (9), i.e. there is no other way to write a term which only contains $\sin (\omega t)$ and other term which contains $\sin (\omega t)$ and is multiplied by $\cos (\omega t)$. A last manipulation can be made to transform the bracket expressions into a power sum of $\sin$ functions, by using the binomial expansion we obtain

10 $$\begin{aligned} \begin{aligned} y(t)&= \left[ a_0 + \sum _{k=1}^\infty \sum _{\begin{array}{c} l=0\\ l=l+2 \end{array}}^k \sum _{m=0}^{l/2} \left( {\begin{array}{c}k\\ l\end{array}}\right) \left( {\begin{array}{c}l/2\\ m\end{array}}\right) (-1)^m \sin ^{2m+k-l}(\omega t) (ab)_{kl} \right] \\&\quad + \left[ \sum _{k=1}^\infty \sum _{\begin{array}{c} l=1\\ l=l+2 \end{array}}^k \sum _{m=0}^{(l-1)/2} \left( {\begin{array}{c}k\\ l\end{array}}\right) \left( {\begin{array}{c}(l-1)/2\\ m\end{array}}\right) (-1)^m\sin ^{2m+k-l}(\omega t) (ab)_{kl} \right] \cos (\omega t) . \end{aligned} \end{aligned}$$

We have obtained an expression which only depends on $\cos (\omega t)$ and the power of $\sin (\omega t)$. Consider now a variable change from $\sin (\omega t)$ to *x*(*t*), where $x(t)\!=\!A_1\sin (\omega t)+A_0$, with $A_0\in \mathbb {R}$, $A_1>0$, and $t \in [0,2\pi /\omega ]$. Its derivative is $x'(t)\!=\!A_1\omega \cos (\omega t)$. By defining the magnitude

11 $$\begin{aligned} A'_1 {:=}&\text {max}(x'(t)) =A_1\omega , \end{aligned}$$

where $\text {max}$ is the maximum function. Finally, Eq. (10) can be transformed into

12 $$\begin{aligned} y(t)&= \underbrace{\left[ a_0 + \sum _{k=1}^\infty \sum _{\begin{array}{c} l=0\\ l=l+2 \end{array}}^k \sum _{m=0}^{l/2} \left( {\begin{array}{c}k\\ l\end{array}}\right) \left( {\begin{array}{c}l/2\\ m\end{array}}\right) (-1)^m \left( \frac{x(t)-A_0}{A_1}\right) ^{2m+k-l} (ab)_{kl} \right] }_{{:=}F\left( \frac{x(t)-A_0}{A_1}\right) } \nonumber \\&\quad + \underbrace{\left[ \sum _{k=1}^\infty \sum _{\begin{array}{c} l=1\\ l=l+2 \end{array}}^k \sum _{m=0}^{(l-1)/2} \left( {\begin{array}{c}k\\ l\end{array}}\right) \left( {\begin{array}{c}(l-1)/2\\ m\end{array}}\right) (-1)^m \left( \frac{x(t)-A_0}{A_1}\right) ^{2m+k-l} (ab)_{kl} \right] }_{{:=}G\left( \frac{x(t)-A_0}{A_1}\right) } \left( \frac{x'(t)}{A_1'}\right) , \end{aligned}$$

where the functions *F* and *G* were defined for further reference. The functional dependence of Eq. (12) resembles that of the first order system defined in Eq. (1), we identify

13 $$\begin{aligned} \begin{aligned} F\!\left( \frac{x(t)-A_0}{A_1} \right)&= f(x(t)) \\ G\!\left( \frac{x(t)-A_0}{A_1} \right) \frac{1}{A_1'}&= g(x(t)) . \end{aligned} \end{aligned}$$

This relationship between Eqs. (1) and (12) can be justified by remembering the uniqueness of Eq. (9) and as a consequence, the uniqueness of Eq. (12). This uniqueness allows us to match Eqs. (1) and (12) based on the same functional dependence, and yielding to Eq. (13). If we force the dynamical variable to satisfy $x(t)\!=\!A_1\sin (\omega t)+A_0$, we can calculate the Fourier analysis of *y*(*t*) to obtain the characteristic curves *f* and *g*. Equation (12) depends on $A_0$, $A_1$, $A_1'$, $a_0$ and $(ab)_{kl}$, where the last two are the Fourier coefficients of *y*(*t*) for $x(t)\!=\!A_1\sin (\omega t)+A_0$, according to Eq. (7). In order to show more explicitly the dependence of the characteristic curves with the parameters of the formalism, we define the set $\{a_k,b_k\}$ of the Fourier coefficients, then, the characteristic curves depend on the parameters as

14 $$\begin{aligned} \begin{aligned} f(x(t))&=f(x(t);\{a_k,b_k\},A_0,A_1) \\ g(x(t))&=g(x(t);\{a_k,b_k\},A_0,A_1,A_1') . \end{aligned} \end{aligned}$$

In summary, the procedure to obtain the system modeling consists of calculating the Fourier coefficients of *y*(*t*) for a sinusoidal dynamical variable $x(t)\!=\!A_1\sin (\omega t)+A_0$, then, by using Eq. (13), we evaluate the *f* and *g* characteristics curves based on the definitions from Eqs. (12) and (13), and finally, we are able to plot the characteristic curves *f* and *g* which represents the system. Then, for the system simulation, we use these characteristic curves for a time-domain simulation. It is important to note that for these simulations the parameters $A_0$, $A_1$, $A_1'$, $a_0$ and $(ab)_{kl}$ remain fixed at the values previously estimated from the sinusoidal response. Since the characteristic curves define the system, we can calculate the driving force $\hat{y}(t)$ that corresponds to other dynamical variable $\hat{x}(t)$ by a simple evaluation of the terms in Eq. (12). In the same way, we can calculate the dynamical variable $\hat{x}(t)$ that corresponds to a given driving force $\hat{y}(t)$ by a direct integration of Eq. (12).

It is worth pointing out some comments:

- The procedure explained above is not the actual practical implementation of the method because there are computationally cheaper alternatives to Eq. (12). The actual procedure is presented in the *Implementation* section.
- We have considered a dynamical variable $x(t)=A_1 \sin (\omega t)+A_0$, but the system modeling is also valid for a function with a phase $x(t)=A_1\sin (\omega t+\phi )+A_0$. In that case, the transformation $t_{new}=t+\phi /\omega$ to both dynamical variable and driving force allows us to use Eq. (12).

#### Fourier analysis and characteristic curves

Before the *Implementation* section, it is important to analyze the terms of Eq. (12) in more detail. In the rest of this section, we found explicit expressions that show the equivalence between the Fourier analysis and the polynomial regressions of the characteristic curves. For convenience of notation, we define

15 $$\begin{aligned} z(t)&{:=}\frac{x(t)-A_0}{A_1} . \end{aligned}$$

By regrouping the terms with the same power in Eq. (12), it is possible to find a Taylor expansion for *F* and *G*, namely

16 $$\begin{aligned} F(z(t))&= \sum _{j=0}^{\infty } f_j (z(t))^j \end{aligned}$$

17 $$\begin{aligned} G(z(t))&= \sum _{j=0}^{\infty } g_j (z(t))^j \end{aligned}$$

where

18 $$\begin{aligned} f_j&=\sum _{\begin{array}{c} k=j\\ k=k+2 \end{array}}^\infty [ab]_{kj} \left[ \sum _{\begin{array}{c} l=k-j\\ l=l+2 \end{array}}^k \left( {\begin{array}{c}k\\ l\end{array}}\right) \left( {\begin{array}{c}l/2\\ (l-k+j)/2\end{array}}\right) \right] \end{aligned}$$

19 $$\begin{aligned} g_j&=\sum _{\begin{array}{c} k=j+1\\ k=k+2 \end{array}}^\infty [ab]_{kj} \left[ \sum _{\begin{array}{c} l=k-j\\ l=l+2 \end{array}}^k \left( {\begin{array}{c}k\\ l\end{array}}\right) \left( {\begin{array}{c}(l-1)/2\\ (l-k+j)/2\end{array}}\right) \right] \end{aligned}$$

and

20 $$\begin{aligned}{}[ab]_{kj}&{:=}\frac{1+(-1)^j}{2}(-1)^{j/2}a_k+\frac{1+(-1)^{j-1}}{2}(-1)^{\frac{j-1}{2}}b_k = {\left\{ \begin{array}{ll} a_k(-1)^{j/2} &{} \text { if } j \text { is even} \\ b_k(-1)^{(j-1)/2} &{} \text { if } j \text { is odd} . \end{array}\right. } \end{aligned}$$

The power series expansions from Eqs. (16) and (17) have a convergence radius of $|z(t)|=|(x(t)-A_0)/A_1|< 1$^70^, therefore the new inputs $\hat{x}(t)$ must be restricted to $A_0-A_1< \hat{x}(t) < A_0+A_1$. By replacing Eq. (20) into Eqs. (18) and (19) we notice that each one of the $f_j$ and $g_j$ coefficients with $j\in \mathbb {N}_0$ depends on the Fourier coefficients $a_j$ and $b_j$ by

21 $$\begin{aligned} f_j=&{\left\{ \begin{array}{ll} f_j(a_j,a_{j+2},\ldots ) &{} \quad \text {if } j \text { is even} \\ f_j(b_j,b_{j+2},\ldots ) &{} \quad \text {if } j \text { is odd} \end{array}\right. } \end{aligned}$$

22 $$\begin{aligned} g_j=&{\left\{ \begin{array}{ll} g_j(a_{j+1},a_{j+3},\ldots ) &{} \text { if } j \text { is even} \\ g_j(b_{j+1},b_{j+3},\ldots ) &{} \text { if } j \text { is odd} . \end{array}\right. } \end{aligned}$$

Notice that $f_j$ and $g_j$ depend on different sets of Fourier coefficients, in particular, $f_j$ depends on the even $a_j$ and odd $b_j$, and $g_j$ depends on the odd $a_j$ and even $b_j$. Moreover, the matrix form of Eq. (18) is an upper triangular matrix, this implies that, if the $f_j$ coefficients are known, then the $a_j$ with *j* even and the $b_j$ with *j* odd can be calculated by back substitution. In analog way, Eq. (19) is an upper triangular matrix, this implies that, if the $g_j$ coefficients are known, then the $a_j$ with *j* odd and the $b_j$ with *j* even can be calculated by back substitution. To illustrate this procedure, let us suppose that we calculate Eqs. (18) and (19) up to $k=4$, then, the matrix form of these equations are

23 $$\begin{aligned} \begin{bmatrix} f_0 \\ f_1 \\ -f_2 \\ -f_3 \\ f_4 \end{bmatrix} =\begin{bmatrix} 1 &{} 0 &{} 1 &{} 0 &{} 1 \\ 0 &{} 1 &{} 0 &{} 3 &{} 0 \\ 0 &{} 0 &{} 2 &{} 0 &{} 8 \\ 0 &{} 0 &{} 0 &{} 4 &{} 0 \\ 0 &{} 0 &{} 0 &{} 0 &{} 8 \\ \end{bmatrix} \begin{bmatrix} a_0\\ b_1 \\ a_2\\ b_3 \\ a_4 \end{bmatrix} \end{aligned}$$

and

24 $$\begin{aligned} \begin{bmatrix} g_0 \\ g_1 \\ -g_2 \\ -g_3 \end{bmatrix} =\begin{bmatrix} 1 &{} 0 &{} 1 &{} 0 \\ 0 &{} 2 &{} 0 &{} 4 \\ 0 &{} 0 &{} 4 &{} 0 \\ 0 &{} 0 &{} 0 &{} 8 \\ \end{bmatrix} \begin{bmatrix} a_1 \\ b_2\\ a_3 \\ b_4 \end{bmatrix} . \end{aligned}$$

Notice that these equations can be separated into the even and odd components

25 $$\begin{aligned} \begin{bmatrix} f_0 \\ -f_2 \\ f_4 \end{bmatrix}= & {} \begin{bmatrix} 1 &{} 1 &{} 1 \\ 0 &{} 2 &{} 8 \\ 0 &{} 0 &{} 8 \\ \end{bmatrix} \begin{bmatrix} a_0\\ a_2\\ a_4 \end{bmatrix} \end{aligned}$$

26 $$\begin{aligned} \begin{bmatrix} f_1 \\ -f_3 \end{bmatrix}= & {} \begin{bmatrix} 1 &{} 3 \\ 0 &{} 4 \\ \end{bmatrix} \begin{bmatrix} b_1\\ b_3 \\ \end{bmatrix} \end{aligned}$$

27 $$\begin{aligned} \begin{bmatrix} g_0 \\ -g_2 \\ \end{bmatrix}= & {} \begin{bmatrix} 1 &{} 1 \\ 0 &{} 4 \\ \end{bmatrix} \begin{bmatrix} a_1\\ a_3 \\ \end{bmatrix} \end{aligned}$$

28 $$\begin{aligned} \begin{bmatrix} g_1 \\ -g_3 \\ \end{bmatrix}= & {} \begin{bmatrix} 2 &{} 4 \\ 0 &{} 8 \\ \end{bmatrix} \begin{bmatrix} b_2\\ b_4 \\ \end{bmatrix} . \end{aligned}$$

These equations can be solved by back substitution as mentioned. The same procedure can be applied for each $k\in \mathbb {N}_0$. The right side in Eqs. (16) and (17) is a polynomial fitting. To visualize this fact, suppose that $x(t)=A_1\sin (\omega t)+A_0$ is a sampled *N*-length sequence, we define $x_n=A_1\sin (2\pi n/N)+A_0$ with $n\in [0,N-1]$, then $(x_n-A_0)/A_1\in [-1,1]$. The discrete version of Eq. (1) is $y_n=f(x_n)+g(x_n) x'_n$. The Fourier coefficients of *y*(*t*) can be obtained from $y_n$ by using the FFT, this is discussed in Supplementary M1. We also define $z_n{:=}(x_n-A_0)/A_1$, $F_n{:=}F(z_n)$ and $G_n{:=}G(z_n)$. Suppose we desire to calculate the polynomial expansion up to order *M*, i.e. $k_{max}=M$, then Eqs. (16) and (17) are written as

29 $$\begin{aligned} F_n&= \sum _{j=0}^{M} f_j z_n^j \end{aligned}$$

30 $$\begin{aligned} G_n&= \sum _{j=0}^{M-1} g_j z_n^j . \end{aligned}$$

It can be expressed in matrix form as

31 $$\begin{aligned} \begin{bmatrix} F_0 \\ F_1 \\ \vdots \\ F_{N-1} \\ \end{bmatrix} = \begin{bmatrix} 1 &{} z_0 &{} z_0^2 &{} \cdots &{} z_0^{M} \\ 1 &{} z_1 &{} z_1^2 &{} \cdots &{} z_1^{M} \\ \vdots &{} \vdots &{} \vdots &{} \cdots &{} \vdots \\ 1 &{} z_{N-1} &{} z_{N-1}^2 &{} \cdots &{} z_{N-1}^{M} \\ \end{bmatrix} \begin{bmatrix} f_0\\ f_1\\ \vdots \\ f_{M}\\ \end{bmatrix} \end{aligned}$$

32 $$\begin{aligned} \begin{bmatrix} G_0 \\ G_1 \\ \vdots \\ G_{N-1} \\ \end{bmatrix} = \begin{bmatrix} 1 &{} z_0 &{} z_0^2 &{} \cdots &{} z_0^{M-1} \\ 1 &{} z_1 &{} z_1^2 &{} \cdots &{} z_1^{M-1} \\ \vdots &{} \vdots &{} \vdots &{} \cdots &{} \vdots \\ 1 &{} z_{N-1} &{} z_{N-1}^2 &{} \cdots &{} z_{N-1}^{M-1} \\ \end{bmatrix} \begin{bmatrix} g_0\\ g_1\\ \vdots \\ g_{M-1}\\ \end{bmatrix} . \end{aligned}$$

The matrices on the right are called Vandermonde matrices. If $F_n$ and $G_n$ are known, then the coefficients of the polynomials are obtained by the inversion of a Vandermonde matrix. If all the $z_n$ terms are different, then Eqs. (29) and (30) are invertible when $(M+1)\le N$ and $M\le N$, respectively. This restricts the maximum order for the polynomial to $M_{max}=N-1$. By defining the Vandermonde matrices from Eqs. (31) and (32) as ${\textbf {Z}}_{M}$ and ${\textbf {Z}}_{M-1}$, we can write

33 $$\begin{aligned} \begin{aligned} \vec {F} =&{\textbf {Z}}_{M} \cdot \vec {f} \\ \vec {G} =&{\textbf {Z}}_{M-1} \cdot \vec {g} , \end{aligned} \end{aligned}$$

and the solutions are found by the least square estimation

34 $$\begin{aligned} \left( {\textbf {Z}}_{M}^T\cdot {\textbf {Z}}_{M}\right) ^{-1} \cdot {\textbf {Z}}_{M}^T \cdot \vec {F} =&\vec {f} \end{aligned}$$

35 $$\begin{aligned} \left( {\textbf {Z}}_{M-1}^T\cdot {\textbf {Z}}_{M-1}\right) ^{-1}\cdot {\textbf {Z}}_{M-1}^T \cdot \vec {G} =&\vec {g} . \end{aligned}$$

Equations (34) and (35) correspond to two polynomial regression problems. Finally, we conclude that Eqs. (16) and (17) show an equivalence between the Fourier analysis and two polynomial fittings. In fact, the FFT algorithms make use of a similar equivalence between the complex Fourier Series and a polynomial expansion, see Ref.^71^ and references therein. See also Supplementary M2, where we discuss the formalism of this work by supposing the dynamical variable *x*(*t*) as a complex function.

However, the association of the $f_j$ and $g_j$ coefficients, which are the coefficients of the polynomial regression of the characteristic curves *f*(*x*) and *g*(*x*) with $x\in (A_0-A_1,A_0+A_1)$ as defined in Eq. (1), to the Fourier coefficients of *y*(*t*) is not reported in literature. Equations (12), (16) and (17) show that the Fourier analysis of $y(t)=f(x(t))+g(x(t))x'(t)$ with $x(t)=A_1\sin {\omega t}+A_0$ is equivalent to the polynomial regression of *f*(*x*) and *g*(*x*) with $x\in ( A_0-A_1, A_0+A_1 )$, moreover, *f* and *g* depend on a different set of Fourier coefficients, therefore they are independent. In the language of circuit theory, by noticing that *f*(*x*) and *g*(*x*) correspond with the characteristic curves of a resistive and a reactive element, respectively, the argument in the last sentence means that in a given first order system, if the reactive element is changed by other one, the Fourier coefficients corresponding to the resistive element must remain the same. In the same way, if the resistive element is changed by other one, the Fourier coefficients corresponding to the reactive element must remain the same.

### Implementation

To implement the method based on experimental or simulation data, we use the fact that continuous time variables are mostly given as their discrete sampled versions where the dynamical variable and the driving force are known only at specific times. For the sake of simplicity, we consider a uniform sampling scheme where the time variables are known each $T_s$ seconds, where $T_s$ is the sampling period. This allows us to obtain the Fourier Series coefficients from the FFT algorithm, as explained in Supplementary M1.

**Figure 1:**
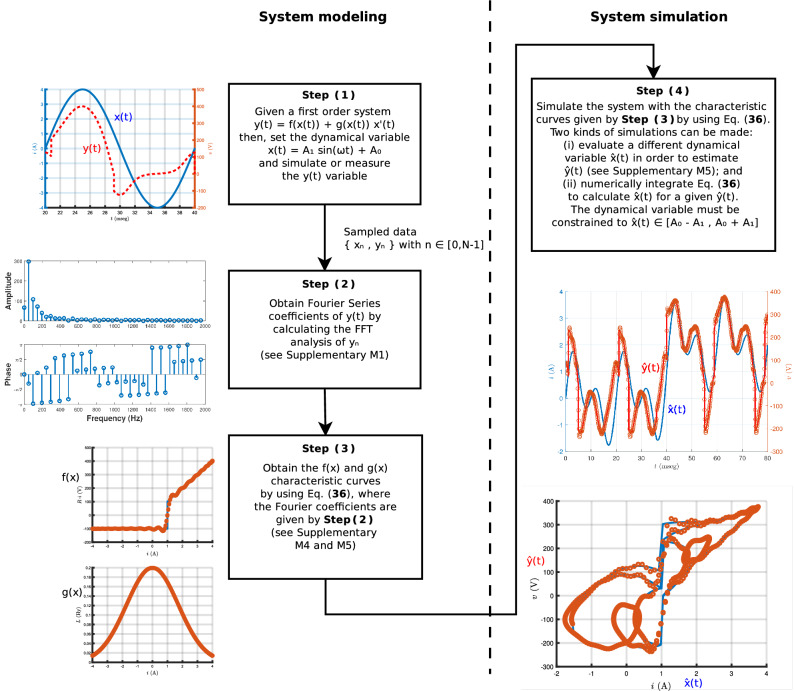
Schematic diagram of the methodology for system modeling and simulating based on the formalism of this work. The illustrations around the boxes show the expected output of each step and have been adapted from an example provided in Supplementary M5. The methodology consists of four main steps which should be applied consecutively: (1) identify the first order equation and set the dynamical variable as a single tone harmonic input; (2) calculate the Fourier series coefficients of the response *y*(*t*) and obtain the amplitude and phase from Fourier analysis; (3) obtain the characteristic curves by evaluating Eq. (36); (4) use the same characteristic curves for a system simulation, where two options are possible: (i) calculate $\hat{y}(t)$ for an arbitrary dynamical variable $\hat{x}(t)$ and not necessarily a single tone; and (ii) calculate $\hat{x}(t)$ for a given $\hat{y}(t)$. In both cases the constraint $\hat{x}(t)\in [A_0-A_1,A_0+A_1]$ must be satisfied.

The procedure for modeling and simulating based on the formalism of this work is represented in Fig. 1. It consists of four steps shown with boxes in the figure. The method can be divided into two main parts: the first one is the estimation of the characteristic curves based on the FFT of the sampled data $\{ x_n, y_n \}$ with $n=[0,N-1]$ and the use of Eq. (36), which is equivalent to Eq. (12) but computationally faster; and the second one is the time-domain simulation by using Eq. (36). In the next paragraph we discuss how this computationally faster equation is obtained and further analysis is given in Supplementary M3. A MATLAB code for the implementation of Eq. (36) is presented in Supplementary M4. The images around the boxes in Fig 1 show the expected output of each step, and are adapted from an example provided in Supplementary M5.

In the rest of the section, we discuss how Eq. (36) is computationally favorable, and then, we present some technical details for each step of the implementation. In the *Formalism* section, we substitute $x(t)=A_1 \sin (\omega t) +A_0$ into Eq. (10) to obtain Eq. (12), this helps us to visualize the connection between the Fourier series and power series, but for computational proposes is favorable to make the substitution into Eq. (9). Then, the following expression is obtained

36 $$\begin{aligned} \begin{aligned} y(t)&= \underbrace{\left[ a_0 + \sum _{k=1}^\infty \sum _{\begin{array}{c} l=0\\ l=l+2 \end{array}}^k \left( {\begin{array}{c}k\\ l\end{array}}\right) \left( 1-\left( \frac{x(t)-A_0}{A_1}\right) ^2\right) ^{l/2}\left( \frac{x(t)-A_0}{A_1}\right) ^{k-l} (ab)_{kl} \right] }_{=f(x(t))} \\&\quad + \underbrace{ \left[ \sum _{k=1}^\infty \sum _{\begin{array}{c} l=1\\ l=l+2 \end{array}}^k \left( {\begin{array}{c}k\\ l\end{array}}\right) \left( 1-\left( \frac{x(t)-A_0}{A_1}\right) ^2\right) ^{(l-1)/2}\left( \frac{x(t)-A_0}{A_1}\right) ^{k-l} (ab)_{kl} \right] \frac{1}{A'_1} }_{=g(x(t))} \, x'(t) , \end{aligned} \end{aligned}$$

where the *f* and *g* characteristic curves have been identified. Equation (36) has double summations instead of Eq. (12) which has triple summations, this implies that they have a computational complexity of $\mathscr {O}(N k_{max}^2)$ and $\mathscr {O}(N k_{max}^3)$, respectively. The details are analyzed in Supplementary M3. This implies that Eq. (36) is computationally favorable and its use is preferred for the practical implementation.

Some technical details of the implementation are presented in the following. We consider that the dynamical variable *x*(*t*) and the driving force *y*(*t*) are given by sampled sequences $x_n$ and $y_n$, with $n=[0,N-1]$. This consideration is based on the fact that in a simulation or data acquisition from an experiment, the time evolution of continuous variables is mostly given by a sampled version of those variables. The continuous time variable can be reconstructed from the sampled data by taking into account the Nyquist–Shannon–Whitaker sampling theorem^72,73^. The sampled data allows us to calculate the Fourier coefficients $a_k$ and $b_k$ from the FFT analysis, allowing us to take advantage of the computationally fast FFT algorithms^74^. A brief account of the relation between the Fourier series and FFT analysis is given in Supplementary M1.

Once the Fourier coefficients of *y*(*t*) are obtained from the FFT analysis, we can make use of Eq. (36) to obtain the *f* and *g* characteristic curves. These characteristic curves define the system, this implies that they do not change even if we consider other dynamical variables or driving forces. This fact can be exploited to simulate other dynamical variables and driving forces by using those characteristic curves. Next, we discuss some technical details of each step of the implementation

Step (1): Set a sinusoidal dynamical variable $x(t)\!=\!A_1\sin (\omega t)+A_0$ into the system in order to simulate or measure the *y*(*t*) response. An illustrative example is shown on the left of the corresponding box in Fig. 1. The *x*(*t*) and *y*(*t*) signals are typically given by sampled sequences $\{ x_n, y_n \}$ with $n \in [0,N-1]$. This signal sampling representation is defined by measuring the value of the continuous function every $T_s$ seconds, where $T_s$ is the sampling period. Consider one period $T=2\pi /\omega$ of the sinusoidal dynamical variable, then the sampled length *N* is defined by $N=T/T_s$, i.e. it represents the amount of points in one period of the sampled sequence.
Step (2): Calculate the Fourier coefficients of *y*(*t*) from the FFT of the vector $\vec {y}=[y_0,y_1,\ldots ,y_{N-1}]$, composed by the sampled sequence $y_n$ of the continuous variable *y*(*t*). The FFT is a fast algorithm^74^ that computes the Discrete Fourier Transform (DFT) with a computational complexity $\mathscr {O}(N \ln {N})$. The result of the DFT is a *N*-length complex vector, which is usually represented by the double-sided amplitude and phase Fourier spectrum. For real sequences, such as is the case for a measurement of a real magnitude $y_n$, it is common to use the representation of the single-sided amplitude and phase Fourier spectrum, as it is shown on the left of step **(2)** in Fig. 1. Supplementary M1 shows the correspondence between the single-sided Fourier spectrum and the Fourier coefficients of *y*(*t*).
Step (3): Evaluate the characteristic curves *f*(*x*) and *g*(*x*) by using Eq. (36), where the parameters are the values of $A_0$, $A_1$, $A_1'=A_1\omega$ and the Fourier coefficients of *y*(*t*) obtained from the sinusoidal dynamical variable. The practical implementation of Eq. (36) requires some $k_{max}$ value for the outer summation, this value is the maximum number of harmonics that will be considered in the analysis. As it is shown in Supplementary M1, the theoretical maximum value for $k_{max}$ is $k_{max}=\lfloor \frac{N}{2}\rfloor - \frac{1+(-1)^N}{2}$. However, as it is analyzed in Supplementary M6, some numerical roundoff errors may appear for $k_{max}>50$. The greater value of $k_{max}$ avoiding these roundoff errors has found to be $k_{max}=40$. However, it is worth to mentioning that by using other numerical techniques to evaluate the terms in the summation, it might be possible to consider even a higher amount of harmonics if necessary. By evaluating the *f*(*x*) and *g*(*x*) characteristic curves based on Eq. (36), we are able to obtain the system modeling, as shown on the left of step **(3)** in Fig. 1.
Step (4): Use Eq. (36) for a time-domain simulation. Two kinds of simulations are proposed: (i) the first one consists on evaluating Eq. (36) for a new known dynamical variable $\hat{x}(t)$ in order to estimate its corresponding driving force $\hat{y}(t)$; (ii) and the second one consists on using a known driving force $\hat{y}(t)$ in order to obtain $\hat{x}(t)$ based on a numerical integration of Eq. (36). In both cases the constraint $\hat{x}(t)\in [A_0-A_1,A_0+A_1]$ must be satisfied. It is important to highlight that only $\hat{x}(t)$ and $\hat{y}(t)$ are modified, but the model parameters must be held fixed to the values obtained from the sinusoidal response of step **(2)**. In order to illustrate the kind (1) of simulation, we present two illustrations below the corresponding box in Fig. 1: the first one shows a given dynamical variable $\hat{x}(t)$ and its corresponding driving force $\hat{y}(t)$ with solid lines, and the computation of the driving force based on Eq. (36) shown by circles; and the second one shows $\hat{y}(t)$ against $\hat{x}(t)$, where the solid lines are the results of the theoretical simulation and the circles corresponds to the predicted values for $\hat{y}(t)$ by using Eq. (36). The details of this illustrative example and a step-by-step implementation of the methodology is presented in Supplementary M5. For completeness, we present some considerations for kind (2) in the following. The basic idea is to use the characteristic curves (which completely define the system), to compute the dynamical variable $\hat{x}(t)$ based on a known driving force $\hat{y}(t)$. By solving Eq. (36) for $\hat{x}'(t)$, we obtain
  37 $$\begin{aligned} \hat{x}'(t)=\frac{\hat{y}(t)-f(\hat{x}(t))}{g(\hat{x}(t))} . \end{aligned}$$
  Equation (37) can be numerically integrated by using, for example, the Euler method^75^
  38 $$\begin{aligned} {\left\{ \begin{array}{ll} \hat{x}(t_0)=x_0 \\ \hat{x}(t_n)=\hat{x}(t_{n-1})+(t_n-t_{n-1})\; \dfrac{\hat{y}(t_{n-1})-f(\hat{x}(t_{n-1}))}{g(\hat{x}(t_{n-1}))} \quad n \ge 1 , \end{array}\right. } \end{aligned}$$
  where $x_0$ is the initial condition and *f* and *g* are defined in Eq. (36). Equation (38) allows us to calculate the dynamical variable $\hat{x}(t)$ from the known driving force $\hat{y}(t)$. If time step $t_n-t_{n-1}$ is not small enough, the use of Eq. (38) may yield to numerical errors. In those cases, a more sophisticated integration method can be employed, such as the Heun’s method^75^
  39 $$\begin{aligned} {\left\{ \begin{array}{ll} \hat{x}(t_0)=x_0 \\ \tilde{x}(t_n)=\hat{x}(t_{n-1})+(t_n-t_{n-1})\; \dfrac{\hat{y}(t_{n-1})-f(\hat{x}(t_{n-1}))}{g(\hat{x}(t_{n-1}))} \\ \hat{x}(t_n)=\hat{x}(t_{n-1})+\dfrac{t_n-t_{n-1}}{2}\; \left( \dfrac{\hat{y}(t_{n-1})-f(\hat{x}(t_{n-1}))}{g(\hat{x}(t_{n-1}))}+\dfrac{\hat{y}(t_{n})-f(\tilde{x}(t_{n}))}{g(\tilde{x}(t_{n}))} \right) \quad n \ge 1 , \end{array}\right. } \end{aligned}$$
  Heun’s method requires two evaluations of Eq. (36) for each integration step and, as a consequence, twice computational time. In the examples presented in Supplementary M5, we have obtained practically the same results by using Eqs. (38) and (39), therefore, only the results from Eq. (38) were presented.

### Applications

The methodology of this work can be applied to any dynamical equation that can be cast into the first order system of Eq. (1). Notice that the left side of Eq. (1) is a time-dependent measurable quantity and the right side depends only on the dynamical variable and its first derivative. A more explicit expression to study the dynamics in Eq. (1) is given by

40 $$\begin{aligned} x'(t)=(y(t)-f(x(t)))/g(x(t)) {:=}F(x(t),y(t)) , \end{aligned}$$

where the function *F* indicates the dependence of $x'(t)$ with *x*(*t*) and *y*(*t*). This equation can be written in a different way by noticing in Eq. (2) that *t* goes in hand with $\omega$, therefore we can define a function $Y(\Omega (t)){:=}y(t)$, where $\Omega (t){:=}\omega t$, then

41 $$\begin{aligned} {\left\{ \begin{array}{ll} x'(t)=F(x(t),Y(\Omega (t))) \\ \Omega '(t)=\omega , \end{array}\right. } \end{aligned}$$

where we have basically transformed the first order system of Eq. (1) into an autonomous second order system. In this section, we firstly discuss in general terms some simple systems which are the nonlinear series RL and parallel RC systems. These two systems are used in Supplementary M5 to present four demonstrative examples: a nonlinear inductor in series with a discontinous resistance, a diode, a diode in parallel with a capacitor, and a nonlinear inductor. Then, in this section, we present a methodology to apply the formalism to a combination of RL and RC systems, where we write the system equations into a convenient form based on two first order equations.

The well known analogies between the electrical components *R*, *L* and *C* and the mechanical analogues *b*, *m* and *k*^34^, and also with other domains such as acoustics, thermal and hydraulics, allows us to apply the methodology as a multidisciplinary tool. More details of these analogies are studied in detail, for example, by the Bond Graph theory^76,77^.

There are many specific potential applications that can be explored with this formalism, in the rest of the section we present only just a few of them in the areas of: electrical measurements, where an hypothetical device called “nonlinear LCR meter” is discussed; viscoelastic materials; and mechanical and structural dynamics, where we discuss a simple example for estimating the nonlinear damping function of a mechanical system.

Further research is needed in order to clarify if the formalism is able to fully represent some important phenomena such as hysteresis and nonlinear resonances. In “Discussion” section, we present a more detailed analysis about the limitations and further research directions in order to elucidate the scope and range of applications of the formalism.

#### Nonlinear series RL system

**Figure 2:**
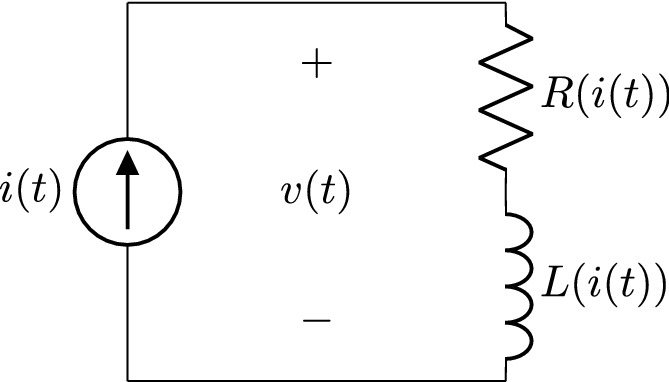
RL series circuit.

We consider the circuit of Fig. 2. On the one hand, if the components are based on linear relationships, the voltage *v* is given by the sum of the resistor and inductor contributions

42 $$\begin{aligned} v(t)=R\;i(t)+L\;i'(t) , \end{aligned}$$

where the constitutive relationships of the resistor $v_R(t)=R\;i_R(t)$ and inductor $v_L(t)=L\;i_L'(t)$ were used. On the other hand, if the components are nonlinear, the resistance and inductance can be considered as current dependent, therefore Eq. (42) changes into

43 $$\begin{aligned} v(t)=R(i(t))\ i(t)+L(i(t))\ i'(t) . \end{aligned}$$

Notice that the nonlinear resistor represents any component that can be defined by a I–V characteristic curve, e.g. a diode or a transistor. Equation (43) presents the same functional dependence as that of Eq. (36). If the dynamical variable is set to $i(t)=A_1\sin (\omega t)+A_0$, then the Fourier series of *v*(*t*) will correspond with Eq. (36), with *y*(*t*) replaced by *v*(*t*) and *x*(*t*) replaced by *i*(*t*). The characteristic curves can be identified as

44 $$\begin{aligned} f(i(t);\{a_k,b_k\},A_0,A_1)=R(i(t))\; i(t) \end{aligned}$$

and

45 $$\begin{aligned} g(i(t);\{a_k,b_k\},A_0,A_1,A_1')=L(i(t)), \end{aligned}$$

where $\{a_k,b_k\}$ are the Fourier coefficients of *v*(*t*) for $i(t)=A_1\sin (\omega t)+A_0$, and $A_1'=\max (i'(t))=A_1\omega$. In brief, based on the Fourier analysis of the response *v*(*t*) from a sinusoidal dynamical variable $i(t)=A_1\sin (\omega t)+A_0$, we have obtained an exact expression for calculating *R*(*i*(*t*)) and *L*(*i*(*t*)). Equation (43) must remain the same functional dependence with any other dynamical variable $\hat{i}(t)$, therefore, the corresponding driving force $\hat{v}(t)$ must verify

46 $$\begin{aligned} \hat{v}(t)=R(\hat{i}(t);\{a_k,b_k\},A_0,A_1)\ \hat{i}(t)+L(\hat{i}(t);\{a_k,b_k\},A_0,A_1,A_1')\ \hat{i}'(t) , \end{aligned}$$

provided that the dynamical variable is restricted to the range $\hat{i}(t)\in [A_0-A_1,A_0+A_1]$.

#### Nonlinear parallel RC system

**Figure 3:**
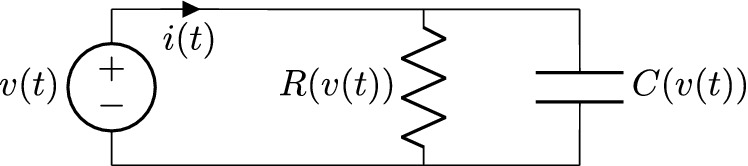
RC parallel circuit.

Consider the circuit of Fig. (3). The constitutive equations for the nonlinear resistor and capacitor can be considered as voltage dependent, $v_R(t)=R(v_R(t))\;i_R(t)$ and $q(t)=C(v_c(t))\;v_c(t)$, respectively. Then, the functional dependence between the dynamical variable *v*(*t*) and the current *i*(*t*) can be written as

47 $$\begin{aligned} i(t)&=i_R(t)+i_C(t) \nonumber \\&= \underbrace{\frac{1}{R(v(t))}}_{{:=}P(v(t))} v(t) + \underbrace{\left( \frac{d C(v(t))}{d v} v(t) + C(v(t)) )\right) }_{{:=}Q(v(t))} v'(t) , \end{aligned}$$

where the chain rule was used in the second term, and the P and Q functions were defined for future reference. If the dynamical variable is set to $v(t)=A_1\sin (\omega t)+A_0$, then the Fourier series of *i*(*t*) will correspond with Eq. (36), with *y*(*t*) replaced by *i*(*t*) and *x*(*t*) replaced by *v*(*t*). In this example, the current corresponds to the driving force and the voltage is the dynamical variable according to the notation of this work. The characteristic curves can be identified as

48 $$\begin{aligned} f(v(t);\{a_k,b_k\},A_0,A_1)=P(v(t))\ v(t) \end{aligned}$$

and

49 $$\begin{aligned} g(v(t);\{a_k,b_k\},A_0,A_1,A_1')=Q(v(t)) , \end{aligned}$$

where $\{a_k,b_k\}$ are the Fourier coefficients of *i*(*t*) for $v(t)=A_1\sin (\omega t)+A_0$, and $A'_1=\max (v'(t))=A_1\omega$. Equation (47) must remain the same functional dependence with any other dynamical variable $\hat{v}(t)$, therefore, the corresponding driving force $\hat{i}(t)$ must verify

50 $$\begin{aligned} \hat{i}(t)=P(\hat{v}(t);\{a_k,b_k\},A_0,A_1)\ \hat{v}(t)+Q(\hat{v}(t);\{a_k,b_k\},A_0,A_1,A_1')\ \hat{v}'(t) , \end{aligned}$$

provided that the dynamical variable is restricted to the range $\hat{v}(t)\in [A_0-A_1,A_0+A_1]$.

#### Combination of RL and RC systems

**Figure 4:**
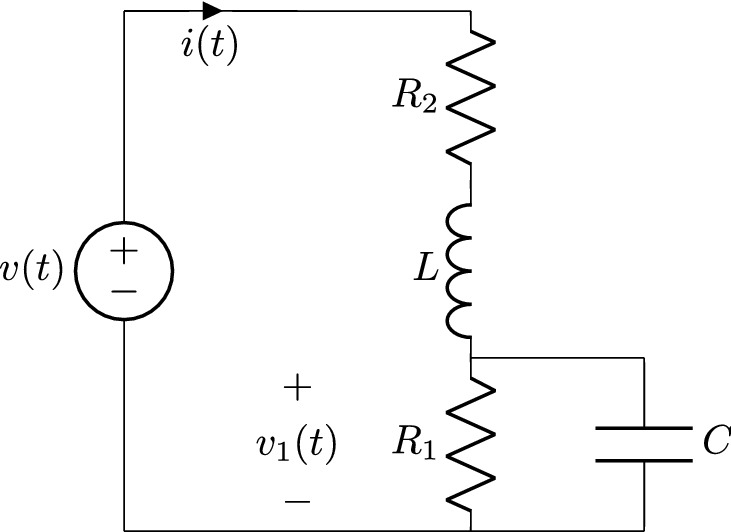
RL series and RC parallel circuit.

A combination of RL and RC systems is shown in Fig. 4. This system has two reactive components L and C, therefore there are two dynamical variables $i(t)=i_L(t)$ and $v_1(t)=v_C(t)$. If all the components are nonlinear, the system equations can be written as two first order nonlinear systems as

51 $$\begin{aligned} v(t)-v_1(t)= & {} R_2(i(t))\; i(t) + L(i(t))\; i'(t) \end{aligned}$$

52 $$\begin{aligned} i(t)= & {} \frac{1}{R_1(v_1(t))}v_1(t)+\left( \frac{dC(v_1(t))}{dv_1} v_1(t)+C(v_1(t)) \right) v_1'(t) \end{aligned}$$

In order to use the formalism of this work for this second order system, it is necessary to model each dynamical variable separately. This means that the subsystem composed of $R_2$ and *L* has to be studied with the methodology explained in section *Nonlinear series RL system*, and the one composed of $R_1$ and *C* has to be studied by the method explained in “Nonlinear parallel RC system”.

By modeling each reactive element L and C as a nonlinear series RL and parallel RC systems, these considerations can be extended to higher order systems. A potential method to be analyzed in future works, which may be considered as a generalization of the linear circuit theory to nonlinear circuit theory, is to analyze higher order systems. The system modeling can be obtained by setting each dynamical variable to a single tone and by calculating the FFT of the corresponding driving force. In summary, we should use the *Nonlinear series RL system* method for each inductor and the *Nonlinear parallel RC system* for each capacitor in the system.

#### Nonlinear LCR meter

The LCR meter basically is a device that applies a sinusoidal single tone to the sample and measures the amplitude and phase of the response in order to obtain a linear parameters of the series RL or parallel RC model of the system at a given fixed frequency. As a direct consequence of sections “Nonlinear series RL system” and “Nonlinear parallel RC system”, it appears the possibility of designing a hypothetical “nonlinear LCR meter”. It is important to mention a well known fact in material science that most electric components and materials are frequency dependent, this implies that the characteristic curves *f* and *g* must dependent on frequency, i.e. $f(x(t);\omega )$ and $g(x(t);\omega )$. Moreover, there is also a device called “impedance analyzer”, which gives us the linear components of the parallel RC or series RL models over a given range of frequencies. The formalism of this work does not include these frequency dependencies. However many of the materials have constant values of their series RL or parallel RC components in a wide range of frequencies, that is the case, for example, of a ferrite-core inductor where the series RL components obtained from an impedance analyzer are approximately constant below the MHz range. To those materials, nonlinear models based on the formalism of this work are expected to correctly describe the system in the range where the parameters are frequency-independent. Further considerations should be carefully addressed to take into account this frequency dependence of the systems, some of them are given in “Discussion” section.

#### Viscoelastic materials

A viscoelastic material has both viscous and elastic properties. The study of viscoelasticity is important in polymer science, biomechanics and biology^78^. Here, we present the use of the formalism for the two simplest models, which are the Maxwell and Kelvin–Voigt models. The Maxwell model is defined by

53 $$\begin{aligned} \frac{d\varepsilon (t)}{dt}=\frac{1}{\eta } \; \sigma (t) + \frac{1}{E} \; \frac{d\sigma (t)}{dt} , \end{aligned}$$

where $\sigma$ is the stress, $\varepsilon$ the strain, *E* the stiffness and $\eta$ the viscosity of the material. The nonlinear equivalent expression of Eq. (53) is given by

54 $$\begin{aligned} \frac{d\varepsilon (t)}{dt}=\frac{1}{\eta (\sigma (t))} \; \sigma (t) + \frac{1}{E(\sigma (t))}\; \frac{d\sigma (t)}{dt} , \end{aligned}$$

where the so called material constants, $\eta$ and *E*, are dependent on $\sigma$. By comparing Eq. (54) with Eq. (1), we identify the driving force $y(t)=d\varepsilon (t)/dt$, the dynamical variable $x(t)=\sigma (t)$, and the characteristic curves $f(x(t))=1/\eta (\sigma (t)) \sigma (t)$ and $g(x(t))=1/E(\sigma (t))$. This implies that, according to Eq. (36), system modeling is obtained by calculating the Fourier analysis of $d\varepsilon (t)/dt$ when the dynamical variable is a single tone, i.e. $\sigma (t)=A\sin {\omega t}+A_0$. Then, we can calculate the characteristic curves and use them to system simulation. According to Fig. 1, two kinds of simulations can be done: (1) calculate the driving force $d\hat{\varepsilon }(t)/dt$ that corresponds to an arbitrary dynamical variable $\hat{\sigma }(t)$; and (2) calculate the dynamical variable based on a known driving force. In both cases, we must take into account the restriction $\hat{\sigma }(t)\in [A_0-A_1,A_0+A_1]$.

The Kelvin–Voigt model is defined by

55 $$\begin{aligned} \sigma (t)=E\, \varepsilon (t)+\eta \; \frac{d\varepsilon (t)}{dt}, \end{aligned}$$

and its nonlinear expression is given by

56 $$\begin{aligned} \sigma (t)=E(\varepsilon (t)) \,\varepsilon (t)+\eta (\varepsilon (t))\; \frac{d\varepsilon (t)}{dt}, \end{aligned}$$

where *E* and $\eta$ depend on strain $\varepsilon$. By comparing Eq. (56) with Eq. (1), we identify the driving force $y(t)=\sigma (t)$, the dynamical variable $x(t)=\varepsilon (t)$, and the characteristic curves $f(x(t))=E(\varepsilon (t))\; \varepsilon (t)$ and $g(x(t))=\eta (\varepsilon (t))$. The system modeling is obtained by calculating the Fourier analysis of $\sigma (t)$ when the dynamical variable is a single tone, i.e. $\varepsilon (t)=A\sin {\omega t}+A_0$. Then, the model can be used for two kinds of simulations: (1) to calculate the driving force $\hat{\sigma }(t)$ that corresponds to an arbitrary dynamical variable $\hat{\varepsilon }(t)$; and (2), to calculate the dynamical variable that corresponds to a known driving force. In both cases, we must take into account the restriction $\hat{\varepsilon }(t)\in [A_0-A_1,A_0+A_1]$.

The present formalism can be used for nonlinear viscoelastic materials or even for linear ones in order to test the range of validity of linear models. Some of the topics that can be studied with the formalism are the large amplitude oscillatory shear (LAOS) obtained from rheological tests^79^ and the dynamical mechanic analysis (DMA) obtained from forced resonance analyzers and free resonance analyzers^80^. Many systems involve a dependency with external parameters such as the frequency or the temperature, see for example Refs.^81–83^. These systems can not be studied in a direct way with the present formalism, see the “Discussion” section for more details.

#### Nonlinear damping

**Figure 5:**
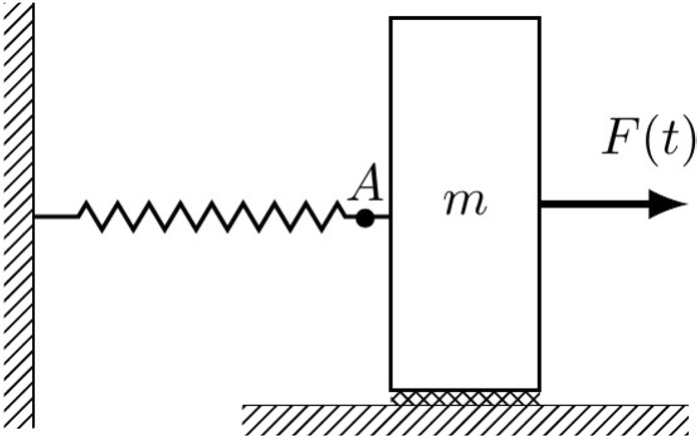
Diagram for a mass subject to a damping and elastic forces.

Consider the diagram of Fig. (5). A body with mass *m* is in contact with a surface that contributes with a damping force $b(v)\, v$. An external force *F*(*t*) is applied to the body which is connected by a spring to a fixed wall. Suppose we measure the force at point *A*, e.g. by using a strain gauge or load cell, the dynamical equation of the system is

57 $$\begin{aligned} F(t)-F_A(t)=b(v(t))\; v(t) +m\; v'(t) , \end{aligned}$$

where $F_A(t)$ is the force measured at point *A*, and *v* is the velocity of the body. By comparing Eq. (57) with Eq. (1), we identify the driving force $y(t)=F(t)-F_A(t)$, the dynamical variable $x(t)=v(t)$, and the characteristic curves $f(x(t))=b(v(t))\;v(t)$ and $g(x(t))=m$. The system modeling is obtained by setting a single tone in the dynamical variable $v(t)=A_1\sin (\omega t)+A_0$ and by calculating the Fourier analysis of the driving force. Once the characteristic curves have been obtained, we can simulate the system with the two kinds of simulations explained in Fig. 1: (1) by calculating the driving force $\hat{F}(t)-\hat{F_A}(t)$ that corresponds to an arbitrary dynamical variable $\hat{v}(t)$; and (2) by calculating the dynamical variable that corresponds to a known driving force. In both cases, we must take into account the restriction $v(t)\in [A_0-A_1,A_0+A_1]$.

Notice that, based on the formalism, the mass is allowed to have a dependence on the velocity of the body. From a practical point of view, we can add this hypothetical dependence *m*(*v*) and follow the complete procedure of Fig. 1. After calculating the characteristic curve *g* with Eq. (36), the expected result should be a constant. Anyway, if necessary, we can force *m* to be a constant. This means that we can calculate only the characteristic curve *f* based on Eq. (36), and set $g(x(t))=m$ for the system simulation. According to the *Formalism* section, the functions *f* and *g* are independent, this means that even if we assume that $g(x(t))=m$ is a constant, the *f*(*x*) characteristic curve is not affected at all.

This simple example allows us to obtain the nonlinear characteristic curve for the nonlinear damping force, and use it for system simulation. It is important to mention that we require to set a single tone in the dynamical variable *v*(*t*) to obtain the system modeling, this may have difficulties for being implemented in a real system, see “Discussion”section for more details. In spite of this limitation, this simple example could be adapted to describe some potential applications in aerospace structures, microelectromechanical systems (MEMS), nonlinear suspension and isolation systems^84–86^.

## Discussion

Linear differential equations can be studied by unified frameworks such as the transfer function (TF) and frequency response function (FRF) in engineering or the Green’s function in physics. In these frameworks, the first harmonic is enough to calculate all possible linear behaviors. However, for nonlinear systems, the presence of higher harmonics can not be avoided. The unified framework for modeling and solving nonlinear differential equations is still under development. Next, we mention some of the advances in this direction and compare them to the formalism of this work.

The Volterra and Wiener series consider that the output is given by the values of the input at previous times. Moreover, the NARMAX model considers that the output is given by both input and output values at previous times, and also the previous and current values of a function that is related to the measurement noise. The formalism of this work differs conceptually from these approaches, because instead of using the previous values to predict the next one, it is focused on calculating the characteristic curves which define the differential equation of the system. In this direction, we use all the input–output data from one complete period of the dynamical variable in order to calculate a polynomial regression for the characteristic curves, and the model only involves the dynamical variable and its first derivative. Therefore, the model do not use the previous values in a explicit way. Conceptually, the amount of values at previous times depends also on the method that is used for calculating the first derivative. If the first derivative is calculated by first-order finite differences, we only require the value of the dynamical variable and the previous one. However, if higher order differentiation methods are used, we will require more than one previous value of the dynamical variable.

The HBM, IBM and their modified versions are mainly based on the transformation of the nonlinear system into an algebraic equation that must be solved for each system separately, for example, by using the Newton-like methods or numerical path continuation^87^. The formalism of this work differs from these approaches mainly because we do not use an explicit definition of the characteristic curves *f* and *g* in any step of the formalism, this allows us to manipulate the first order equation solving it beforehand, and as a result, Eq. (36) remains valid for all the systems that are based on a first order differential equation such as Eq. (1). This formalism, as shown in Supplementary M3, has a low computational complexity, and this allows us to consider up to the 40th harmonics, as shown in Supplementary M5, which is remarkably higher than the harmonics that are usually considered in the bibliography. It is worth mentioning that more research is needed to elucidate whether the formalism is able to fully describe phenomena such as nonlinear resonances, periodic and quasi-periodic oscillations, bifurcations and limit cycles, which are the main applications of HBM and DFs^88,89^. Additional considerations are given at the end of this section.

The OFRF^49–51^ and HOSIDFs^52^ methods are important contributions to the study of nonlinear system modeling based on the frequency domain. Both methods provide extensions of the well known methods of TF and FRF, which are widely used in linear system theory, into nonlinear systems. In particular, the OFRF presents a recursive method to obtain the coefficients of a nonlinear differential equation from the Fourier analysis, which requires many simulations and algebraic operations for each system under study. The HOSIDFs defines a virtual harmonic generator and many describing functions up to *nth* order, where each describing function is associated to an amplitude-dependent transfer function. The formalism that is presented in this work differs conceptually from the OFRF and HOSIDFs methods, because it does not search an extension of the TF and FRF into nonlinear systems, instead it aims to the search of a direct relation between the Fourier coefficients and the characteristic curves that define the nonlinear system. By using this approach, we avoid the direct use of GFRFs and virtual harmonic generators, instead, we manipulate the Fourier series beforehand in order to represent a nonlinear first order system. Even though the SIRPS method presented in this work is conceptually different from the OFRF and HOSIDFs methods, all these formalisms are expected to be related in some way, and this work may be considered as a contribution in order to define a unified framework to study nonlinear systems in the frequency domain.

The formalism of this work is intuitively based on a basic result from linear RL (RC) systems, where the knowledge of the amplitude and phase of the first harmonic allows us to find the values of R and L (R and C). We have extended this concept to nonlinear RL (RC) systems, by showing the equivalence between the Fourier coefficients of the driving force and the polynomial regressions of the characteristic curves of a first order differential equation. This result explicitly shows that the Fourier series can represent two independent nonlinear characteristic curves *f*(*x*) and *g*(*x*). This is a different perspective compared with the usual interpretation of the Fourier series as composed by $\sin$ and $\cos$ functions that form an orthogonal countable basis for square-integrable functions on a bounded domain. This perspective is a direct consequence of the formalism, and may lead to a reinterpretation of the Fourier series in the field of differential equations. Moreover, as shown in Supplementary M5, and based on the *Formalism* section, the implementation of the formalism presents both characteristics of Fourier and polynomial regression in a combined form, and therefore both the Runge phenomenon^90^ and the Gibbs-Wilbraham phenomenon^91–93^ are present simultaneously. These remarks can be a starting point to generate further research into those directions.

A comment can be said about the computational time from Supplementary M3. On the one hand, the model is obtained from FFT, which has a computational complexity of $\mathscr {O}(N\ln N)$^74^, where *N* is the length of the sequence $y_n$. On the other hand, the simulation by using Eq. (36) has a computational complexity of $\mathscr {O}(N k_{max}^2)$, which is $\mathscr {O}(N)$ for a fixed $k_{max}$ value. Due to this low computational complexity in both modeling and simulation, this technique is expected to be suitable for real-time applications, for example, for being implemented in an embedded system. Moreover, there may also be some consequences in the formalism of control theory.

A limitation can be made concerning to the range of the dynamical variable, if the Fourier coefficients are calculated by using a dynamical variable $x(t)=A_1\sin (\omega t)+A_0$, which is restricted to the range $[A_0-A_1,A_0+A_1]$, then, any other dynamical variable $\hat{x}(t)$ used for simulation must be restricted to the same range. This limitation appears because the Fourier series is expressed as a power series expansion, which presents a divergence when the dynamical variable $\hat{x}$ goes out of this range.

Another limitation may appear for some real systems. The procedure explained in this work requires being able to set a single tone for the dynamical variable in order to obtain the characteristic curves. But for some real systems due to equipment limitations or simply due to limitations in the process, we may not be able to use a single sinusoidal tone to obtain the characteristic curves. Further research can be addressed in order to overcome this limitation.

A consideration can be made concerning the application of this formalism to some real systems that have characteristic curves *f* and *g* which depend on the frequency, i.e. $f(x(t);\omega )$ and $g(x(t);\omega )$, in fact, that is the case of virtually all materials in material science. This has been briefly discussed in the application *Nonlinear LCR meter*. A detailed study must be addressed in order to analyze this frequency dependence of the systems and the possibility of being described by the formalism presented here. We discuss some considerations to this direction in the following. Consider that the characteristic curves are frequency dependent, we can add the $\omega$ variable as a parameter of the characteristic curves $f(x(t);\omega )$ and $g(x(t);\omega )$. Moreover, notice that in the formalism, we use a dynamical variable $x(t)=A_1\sin (\omega t)+A_0$ to obtain the system modeling, that implies that the nonlinear model is strictly valid for $f(x(t);\omega )$ and $g(x(t);\omega )$. If the dynamical variable is changed to $\hat{x}(t)=A_1\sin (\hat{\omega } t)+A_0$, with $\hat{\omega }\ne \omega$, then, we would strictly obtain another characteristic curves $f(x(t),\hat{\omega })$ and $g(x(t),\hat{\omega })$. The discrepancies in these characteristic curves are expected to be small when $\hat{\omega }$ is close to $\omega$ and larger when they are far away. If we consider a set of frequencies $\omega _i$ with $i\in [1,n]$ for the dynamical variable, then we would be able to apply the formalism for each frequency obtaining a set of characteristic curves {$f_i(x(t),\omega _i),g_i(x(t),\omega _i)$} with $i\in [1,n]$, where each model is valid for a range around its corresponding frequency. It is important to mention that the characteristic curves in this work are obtained when the dynamical variable is a single tone. If the characteristic curves are not frequency dependent, we can assure that the model is valid for an arbitrary driving force, in particular it is valid for a single tone. However, in the case of frequency dependent characteristic curves there is an additional problem to address, which can be formulated as: for a given single tone of the driving force, which are the frequencies that are present in the dynamical variable?. This is a non-trivial problem that may be addressed in future works. Additionally, most of the materials also depend with other external parameters, such as temperature or power dissipation. The formalism does not have these effects into account, and further research could be investigated in order to include them.

Finally, further research is needed to elucidate if the formalism is able to fully represent other complex phenomena such as bifurcations, hysteresis effects, and also some other important phenomena that are intrinsically related to second or higher order differential equations, such as chaos in dynamical systems^94^ and nonlinear resonances^95^.

## Conclusions

Clear evidence is found on the equivalence between Fourier analysis of the driving force *y*(*t*) and polynomial regressions of the characteristic curves *f*(*x*) and *g*(*x*) for a first order system $y(t)=f(x(t))+g(x(t))\,x'(t)$. The expressions of this work allow us to obtain the system modeling based on the sinusoidal dynamical variable $x(t)=A_1\sin (\omega t)+A_0$ and by the Fourier analysis of its corresponding driving force *y*(*t*). The model consists of the Fourier coefficients $a_k$ and $b_k$, and the values of $A_0$, $A_1$ and $A_1'=A_1\omega$. Then, this model can be used for a time-domain simulation with the restriction $\hat{x}\in [A_0-A_1,A_0+A_1]$. In the language of circuit theory, *f*(*x*) and *g*(*x*) correspond to resistive and reactive nonlinear elements, respectively. Therefore, the formalism presented here allows us to relate the Fourier analysis of the driving force to the characteristic curves of these two nonlinear elements. The characteristic curves depend on a different set of Fourier coefficients, this allows us to identify each function independently.

The practical implementation of the method is based on that the variables *x*(*t*) and *y*(*t*) are usually given by the sampled sequences {$x_n$} and {$y_n$} with $n\in [0,N-1]$, this allows us to obtain the Fourier coefficients from the FFT and, as a consequence, the entire methodology has a computational complexity small enough for real-time applications. Furthermore, the formalism allows us to consider a higher amount of harmonics in the system modeling than is usually reported. Some demonstrative examples are presented by considering up to 40ths harmonics.

## Supplementary Information

Supplementary Information.

## Data Availability

The data that support the present study are available from the corresponding author upon reasonable request.

## Code availability

MATLAB codes used for the present study are available from the corresponding author upon reasonable request.
