# Supplementary material for: Determination of the characteristic curves of a nonlinear first order system from Fourier analysis
Source: Sci Rep. 2023 Feb 2;13:1955. doi: 10.1038/s41598-023-29151-5 (PMC9895081; doi:10.1038/s41598-023-29151-5)
Supplement: Supplementary file 1 — Supplementary Information. [file 41598_2023_29151_MOESM1_ESM.pdf]

(Supplementary material)
Determination of the characteristic curves of a nonlinear first order system from Fourier analysis

Federico J. Gonzalez[1,*]

[1]Instituto de Física Rosario (CONICET-UNR), Bv. 27 de Febrero 210 Bis, S2000EZP Rosario, Argentina
[1]Facultad de Ciencias Exactas, Ingeniería y Agrimensura (UNR), Av. Pellegrini 250, S2000BTP Rosario, Argentina
[*]fgonzalez@ifir-conicet.gov.ar

Supplementary M1. Relation between FFT and Fourier Series

The Fast Fourier Transform (FFT) is an algorithm that calculates the Discrete Fourier Transform (DFT) of a sequence. The DFT transforms a sequence of N complex numbers $\{x_n\} := \{x_0, x_1, \cdots, x_{N-1}\}$ into another sequence of complex numbers $\{X_k\} := \{X_0, X_1, \cdots, X_{N-1}\}$, which is defined by[1]

$$X_k = \sum_{n=0}^{N-1} x_n e^{-i\frac{2\pi}{N}kn}, \tag{1}$$

with $k \in [0, N-1]$. The Inverse Discrete Fourier Transform (IDFT) is obtained by

$$x_n = \frac{1}{N}\sum_{k=0}^{N-1} X_k e^{i\frac{2\pi}{N}kn}, \tag{2}$$

with $n \in [0, N-1]$. Consider a real function $x(t)$ with period T and $t \in \mathbb{R}$, it can be expressed as a Fourier Series (FS)

$$x(t) = a_0 + \sum_{k=1}^{\infty} \left(a_k \cos(k\omega t) + b_k \sin(k\omega t)\right) . \tag{3}$$

If $x(t)$ is sampled with period T_s, its sampled sequence $x_n = x(t_n = nT_s)$ and the sequence length N is chosen to verify $x(t_0) = x(t_0 + NT_s)$, then by using the Whittaker-Nyquist-Shannon sampling theorem[2], the original signal $x(t)$ can be fully reconstructed by the expression

$$x_n = x(t_n) = x(nT_s) = a_0 + \sum_{k=1}^{\lfloor \frac{N}{2} \rfloor - \frac{1+(-1)^N}{2}} \left(a_k \cos\left(k\frac{2\pi}{N}n\right) + b_k \sin\left(k\frac{2\pi}{N}n\right)\right) , \tag{4}$$

where $\lfloor\rfloor$ is the floor function. Note that the maximum frequency is

$$f_{max} = \frac{k_{max}}{T} = \frac{\lfloor \frac{N}{2} \rfloor - \frac{1+(-1)^N}{2}}{N} f_s < \frac{1}{2} f_s , \tag{5}$$

where $T = NT_s$ and $f_s = 1/T_s$ is the sampling frequency. The last inequality can be obtained by separating the cases

$$\frac{\lfloor \frac{N}{2} \rfloor - \frac{1+(-1)^N}{2}}{N} = \begin{cases} \frac{1}{2} - \frac{1}{N} & \text{if } N \text{ is even} \\ \frac{1}{2} - \frac{1}{2N} & \text{if } N \text{ is odd} . \end{cases} \tag{6}$$

Equation (5) is consistent with the sampling Shannon condition $f_s > 2f_{max}$ for a fully reconstructed signal.

In the following, we will find the relation between the Fourier coefficients of Eq. (4) with those of Eq. (2).

For real sequences x_n it is verified

$$X_k^* = X_{N-k} , \tag{7}$$

with $k \in [1, \lfloor \frac{N}{2} \rfloor - \frac{1+(-1)^N}{2}]$, where * denotes the complex conjugate, and $\lfloor\rfloor$ is the floor function. This result follows directly from Eq. (1) by using the relation $x_n = x_n^*$, which is valid for real sequences. By using Eq. (7), we can expand Eq. (2) as

$$\begin{aligned} x_n &= \frac{X_0}{N} + \sum_{k=1}^{\lfloor \frac{N}{2} \rfloor - \frac{1+(-1)^N}{2}} \left(\frac{X_k}{N} e^{i\frac{2\pi}{N}kn} + \frac{X_k^*}{N} e^{-i\frac{2\pi}{N}kn}\right) + \frac{(-1)^n(1+(-1)^N)}{2N} X_{\lfloor \frac{N}{2} \rfloor} \\ &= \frac{X_0}{N} + \frac{1}{N}\sum_{k=1}^{\lfloor \frac{N}{2} \rfloor - \frac{1+(-1)^N}{2}} 2\Re\mathfrak{e}(X_k e^{i\frac{2\pi}{N}kn}) + \frac{(-1)^n(1+(-1)^N)}{2N} X_{\lfloor \frac{N}{2} \rfloor} . \end{aligned} \tag{8}$$

By replacing $X_k = \mathfrak{Re}(X_k) + i\mathfrak{Im}(X_k)$ into Eq. (8) we obtain

$$x_n = \frac{X_0}{N} + \sum_{k=1}^{\lfloor \frac{N}{2} \rfloor - \frac{1+(-1)^N}{2}} \frac{2\mathfrak{Re}(X_k)}{N} \cos\left(\frac{2\pi}{N}kn\right) - \sum_{k=1}^{\lfloor \frac{N}{2} \rfloor - \frac{1+(-1)^N}{2}} \frac{2\mathfrak{Im}(X_k)}{N} \sin\left(\frac{2\pi}{N}kn\right) + \frac{(-1)^n(1+(-1)^N)}{2N} X_{\lfloor \frac{N}{2} \rfloor} . \tag{9}$$

Finally, by comparing Eqs. (9) and (4) we identify

$$a_k = \begin{cases} \frac{X_0}{N} = \frac{\mathfrak{Re}(X_0)}{N} & \text{if } k = 0 \\ 2\frac{\mathfrak{Re}(X_k)}{N} & \text{if } k > 0 \end{cases} \tag{10}$$

and

$$b_k = \begin{cases} 0 & \text{if } k = 0 \\ -2\frac{\mathfrak{Im}(X_k)}{N} & \text{if } k > 0 \, . \end{cases} \tag{11}$$

Most of the numerical FFT methods calculate the double-sided DFT, which is consistent with the definition of Eq. (1). In order to calculate single-sided DFT, we use Eqs. (10) and (11).

A Matlab script to obtain the single-sided FS coefficients from the FFT function is presented here

```
fftx=fft(x); % calculate the FFT of the vector x
N=length(x);
P2=fftx/N; % double-sided FFT
P1=P2(1:N/2+1); % single-sided FFT
P1(2:end-1) = 2*P1(2:end-1);
ak=real(P1); % Fourier coefficients ak and bk
bk=-imag(P1); % obtained from the single-sided Fourier spectrum
```

Supplementary M2. Complex dynamical variable

The equations which were deduced in this work are based on a real dynamical variable $x(t)$, which is forced to be equal to $x(t) = A_1 \sin \omega t + A_0$ for the system modeling. This appendix considers an extension to the complex domain, where the dynamical variable is a complex function $\tilde{x}(t)$. For the system modeling, consider that it verifies $\tilde{x}(t) = re^{i\omega t} + \tilde{A}_0$, with $r > 0$ and $\tilde{A}_0$ a complex number. As it is shown below, the mathematical expressions which arise in this complex formalism are simpler compared to the real formalism. However, the real formalism which is used throughout this work can be more directly applied to experimental or simulation data. Suppose that $\tilde{y}(t)$ is a complex-valued function of a real variable t with period T, we can expand it with the complex Fourier series

$$\tilde{y}(t) = \sum_{k=-\infty}^{\infty} C_k e^{ik\omega t} \, , \tag{12}$$

where $\omega = 2\pi/T$ is the fundamental frequency and

$$C_k = \frac{\omega}{2\pi} \int_0^{2\pi/\omega} \hat{y}(t) e^{-ik\omega t} \, . \tag{13}$$

By replacing $C_k = \mathfrak{Re}(C_k) + i\mathfrak{Im}(C_k)$ into Eq. (12), we obtain

$$\tilde{y}(t) = \sum_{k=-\infty}^{\infty} \mathfrak{Re}(C_k) e^{ik\omega t} + i \sum_{k=-\infty}^{\infty} \mathfrak{Im}(C_k) e^{ik\omega t} \, . \tag{14}$$

By using $\tilde{x}(t) = re^{i\omega t} + \tilde{A}_0$ and $\tilde{x}'(t) = i\omega re^{i\omega t}$, Eq. (14) can be rewritten as

$$\tilde{y}(t) = \sum_{k=-\infty}^{\infty} \mathfrak{Re}(C_k) \left(\frac{\tilde{x}(t) - \tilde{A}_0}{r}\right)^k + \sum_{k=-\infty}^{\infty} \mathfrak{Im}(C_k) \left(\frac{\tilde{x}(t) - \tilde{A}_0}{r}\right)^{k-1} \frac{\tilde{x}'(t)}{\tilde{A}'_1} \, , \tag{15}$$

where $\tilde{A}'_1 := \tilde{A}_1 \omega$. In this complex version, the polynomial expansion analog to Eqs. (**16**) and (**17**) of the main text are in fact decoupled, and can be directly identified as

$$\begin{aligned} f_k &= \mathfrak{Re}(C_k) \\ g_k &= \mathfrak{Im}(C_{k+1}) \, . \end{aligned} \tag{16}$$

In the following, we consider the discrete version of the complex formalism, which may be useful for a practical implementation based on a complex sampled data $\{\tilde{x}_n, \tilde{y}_n\}$ for $n \in [0, N-1]$. As a result of this analysis, we obtain the relation between the complex Fourier series and the FFT of the complex function $\tilde{y}_n$.

Consider a sequence of N complex numbers $\tilde{y}_n := \{\tilde{y}_0, \tilde{y}_1, \cdots, \tilde{y}_{N-1}\}$, the Inverse Discrete Fourier Transform (IDFT) is (see the context from Supplementary M1, Eq. (2))

$$\tilde{y}_n = \frac{1}{N} \sum_{k=0}^{N-1} Y_k e^{i \frac{2\pi}{N} kn}, \tag{17}$$

with $n \in [0, N-1]$. By replacing $Y_k = \mathfrak{Re}(Y_k) + i\mathfrak{Im}(Y_k)$ into this equation, we obtain

$$\tilde{y}_n = \sum_{k=0}^{N-1} \frac{\mathfrak{Re}(Y_k)}{N} \left(e^{i \frac{2\pi}{N} n}\right)^k + i \sum_{k=1}^{N-1} \frac{\mathfrak{Im}(Y_k)}{N} \left(e^{i \frac{2\pi}{N} n}\right)^k. \tag{18}$$

By using the discrete dynamical function $\tilde{x}_n = r e^{i \frac{2\pi}{N} n} + \tilde{A}_0$ and its complex differentiation $\tilde{x}'_n = i(2\pi r) e^{i \frac{2\pi}{N} n}/N$ into Eq. (18), we obtain

$$\tilde{y}_n = \left(\sum_{k=0}^{N-1} \frac{\mathfrak{Re}(Y_k)}{N} \left(\frac{\tilde{x}_n - \tilde{A}_0}{r} \right)^k \right) + \left(\sum_{k=0}^{N-2} \frac{\mathfrak{Im}(Y_{k+1})}{N} \left(\frac{\tilde{x}_n - \tilde{A}_0}{r} \right)^k \right) \frac{\tilde{x}'_n}{|\tilde{x}'_n|}, \tag{19}$$

where $|\ |$ denotes the complex modulus and $|\tilde{x}'_n| = (2\pi r)/N$. Equation (19) is the complex analog to Eq. (**12**) of the main text. In this complex version, the polynomial expansions analog to Eqs. (**15**) and (**16**) of the main text are given by

$$\begin{aligned} f_k &= \frac{\mathfrak{Re}(Y_k)}{N} && \text{if } k \in [0, N-1] \\ g_k &= \frac{\mathfrak{Im}(Y_{k+1})}{N} && \text{if } k \in [0, N-2]. \end{aligned} \tag{20}$$

Supplementary M3. Computational complexity

On the one hand, the model is obtained from FFT, which has a computational complexity of $\mathscr{O}(N \ln N)$[1], where N is the length of the sequence y_n. On the other hand, the simulation by using Eq. (**12**) of the main text has a computational complexity of $\mathscr{O}(N k_{max}^3)$, which is $\mathscr{O}(N)$ for a fixed k_{max} value. This computational complexity can be reduced to $\mathscr{O}(N k_{max}^2)$ by using Eq. (**36**) of the main text instead of Eq. (**12**). To visualize this fact, we evaluate the computational time vs. k_{max} by using Eqs. (**36**) and (**12**) for $N = 2000$, the results are shown in supplementary figure 1. We can observe the k_{max}^2 trend for Eq. (**36**). We also evaluate the computational time vs. N for $k_{max} = 40$, this is shown in supplementary figure 2. The linear trend can be observed for both equations. Based on this analysis, we can conclude that both estimation and simulation procedures can be calculated by a low computationally technique, then it is expected to be suitable for real-time applications.

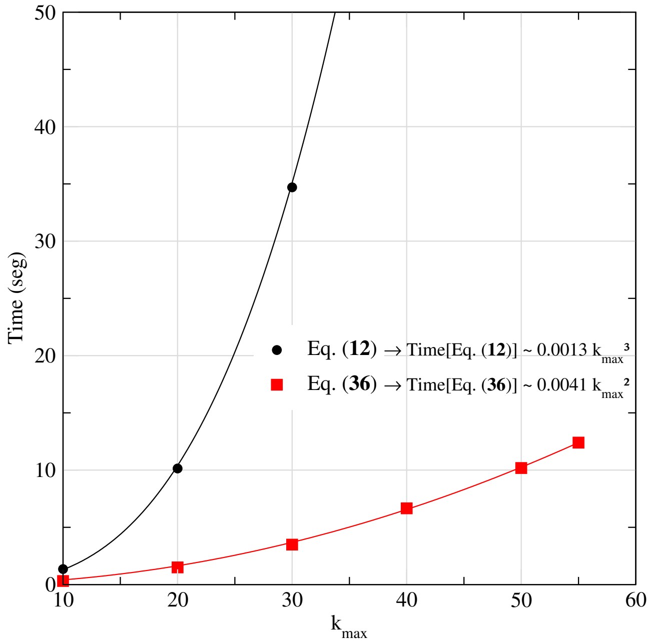

Supplementary figure 1. Computational time vs. k_{max} for Eqs. (**12**) and (**36**) of the main text by using an Intel® Core™ i3-2nd generation processor. The value $N = 2000$ remains fixed.

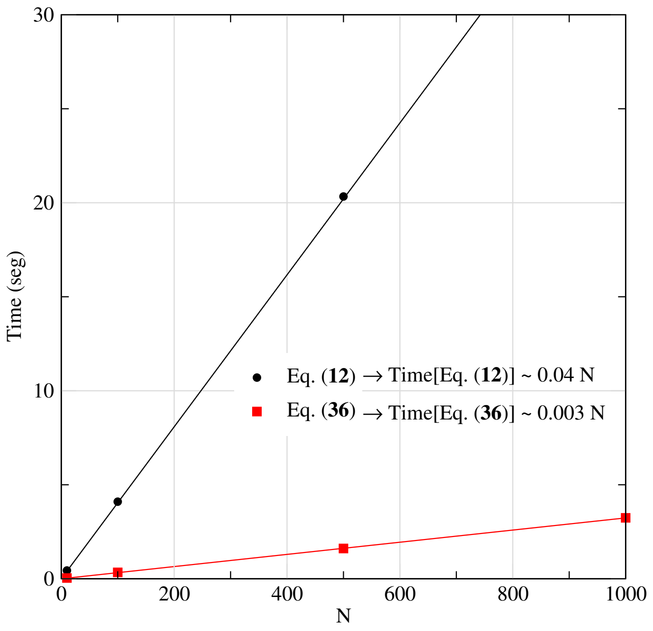

Supplementary figure 2. Computational time vs. N for Eqs. (**12**) and (**36**) of the main text by using an Intel® Core™ i3-2nd generation processor. The value $k_{max} = 40$ remains fixed.

Supplementary M4. Matlab script

A simplified implementation of the f function from Eq. (**36**) of the main text is presented below. The implementation of the g function is analog.

```
xind=2; % starting index for x
ix=0; % ix=index
Nstep=10; % number of steps
kmax=40; % maximum value of the outer summation
while (xind<=length(x)) % iterates over the dataset points within the range [0,N
    -1]
 ix=ix+1;
 x(ix)=x(xind);
 F(ix)=ak(1);
 y(ix)=y(xind);
 for k=1:kmax % outer summation
  l=0;
  while(l<=k) % inner summation
   abk=ak(k+1)*cos(pi/2*(k-l))+bk(k+1)*sin(pi/2*(k-l));
   F(ix)=F(ix)+nchoosek(k,l)*(1-((x(ix)-A0)/x0)^2)^(1/2)*((x(ix)-A0)/x0)^(k-l)*
       abk;
   l=l+2;
  end
 end
 xind=xind+Nstep;
end
```

Supplementary M5. Demonstrative examples

This section presents some demonstrative examples of the formalism of this work. We first analyze a nonlinear inductor in series with a discontinuous resistance in order to show the complete procedure. Then we consider more simple and illustrative examples in order to discuss some specific details of the formalism. We will follow the steps that are presented in figure **1** of the main text.

Nonlinear inductor in series with a discontinuous resistance

In this example, we consider a nonlinear RL system with a discontinuity, where the R and L components are defined by

$$R(i) = \begin{cases} -100/i & \text{if } i \leq 1 \text{ A} \\ 100 & \text{if } i > 1 \text{ A} \end{cases} \tag{21}$$

$$L(i) = 0.2\, e^{-i^2/6} \,. \tag{22}$$

According to the section *Nonlinear series RL system* of the main text, the dynamical variable for this system is the current $i(t)$, the driving force is the voltage $v(t)$, and the system satisfies the first order differential equation

$$v(t) = R(i(t))\; i(t) + L(i(t))\; i'(t) \,. \tag{23}$$

The characteristic curves are $f(i) = R(i) \cdot i$ and $g(i) = L(i)$. In order to obtain the system modeling, we use a dynamical variable $i(t) = A_1 \sin(\omega t)$, with $A_1 = 4$ A and $\omega = 2\pi 50$ rad/seg. The dynamical variable $i(t) = A_1 \sin(\omega t)$ and the driving force $v(t)$ are shown in supplementary figure 7. This step corresponds to Step **(1)** in figure **1** of the main text. The dataset for the system modeling is obtained with a simulation step $T_s = 10$ μseg and a frequency $f = 50$ Hz. Therefore, the fundamental period is $T = 1/f = 20$ $mseg$ and the sequences $\{x_n\}$ and $\{y_n\}$ have a length $N = T/T_s = 2000$. Now, we calculate the Fast Fourier Transform (FFT) of the response $v(t)$, according to Step **(2)** in figure **1** of the main text. The direct result of the FFT is the double-sided spectrum of y_n. For real signals, as it is the case of y_n, we can calculate the single-sided FFT from the double-sided FFT, see Supplementary M1. The single-sided FFT for this demonstrative example is shown in supplementary figure 4. The maximum number of harmonics that are computed by this method is $k_{max} = \lfloor N/2 \rfloor - (1 + (-1)^N)/2$, supplementary figure 4 only shows up to the 40th harmonic. It can be seen from the figure that the amplitude at 2000 Hz, which corresponds with the 40th harmonic, is at least 100 times smaller than the first harmonic. This indicates that a model with 40 harmonics is probably enough to fully capture the dynamics. Then, the Fourier coefficients can be calculated by using

$$\begin{aligned} a_k &= |P_k| \cos(\angle P_k) \\ b_k &= -|P_k| \sin(\angle P_k) \,, \end{aligned} \tag{24}$$

where P_k is the single-sided FFT of the signal and the sub-index identifies the number of harmonic, which in this example will be constrained to $k \in [0, 40]$. Another equivalent form to obtain the Fourier coefficients is from the real and imaginary parts of the FFT single-sided spectrum, by using Eqs. (10) and (11) from Supplementary M1, see also the Matlab script at the end of that Supplementary. In this example we obtain a total of $2 \cdot 40 + 1 = 81$ Fourier coefficients for the system modeling. It is worth mentioning that this simulations is noiseless, but in case of noise presence, use can be made of the wide range of techniques of spectral density estimation[3] in order to obtain a cleaner Fourier spectrum. The next step is Step **(3)** in figure **1** of the main text, it corresponds to the computation of the characteristic curves. We use Eq. (**36**) of the main text to compute the characteristic curves $f(i) = R(i) \cdot i$ and $g(i) = L(i)$. The explicit dependence with the model parameters can be expressed as $f(i; \{a_k, b_k\}_{k \in [0,40]}, A_0 = 0, A_1 = 4)$ and $g(i; \{a_k, b_k\}_{k \in [0,40]}, A_0 = 0, A_1 = 4, A_1' = 400\pi)$. Supplementary figures 5 and 6 show the R and L components by evaluating Eq. (**36**) in comparison to the theoretical curves of Eqs. (21) and (22), respectively. For a good visualization of the figures, Eq. (**36**) has been calculated each 10 data points, i.e. the figures have 2000/10=200 circles each one. Supplementary figure 5 shows an oscillatory effect at the discontinuity that is typical of a Gibbs-Wilbraham phenomenon[4–6]. In order to quantitatively assess the error between the theoretical simulation and the model, we compute the root-mean-square error (RMSE). We obtain RMSE $= 9.66$ for the f function and RMSE $= 1 \times 10^{-5}$ for the g function. The g curve has been correctly identified and there is no presence of the discontinuity, this is a consequence of that f and g depends on different Fourier coefficients, as shown in Eqs. (**21**) and (**22**) of the main text. For illustrative purposes, we show the comparison of the I-V curves from the simulation for the sinusoidal response and the points computed by Eq. (**36**) in supplementary figure 7. We obtain RMSE $= 9.66$ in agreement with the error of the f function. The Gibbs phenomenon at the discontinuity represents the main contribution to the error. For completeness, we have calculated the V-I curves for different values of the maximum number of harmonics, this is shown in supplementary figure 8. As mentioned above, we have chosen up to the 40th harmonic, i.e. $k_{max} = 40$.

At this stage, we have estimated the characteristic curves f and g. In order to test the model, we consider a simulation according to Step (**4**) in figure **1** of the main text. In a first test, we address the kind (i) of simulation by setting a dynamical variable $\hat{i}(t) = \sin(2\pi 50\; t) + \sin(2\pi 100\; t) + 2\Theta(t - 2/50)$ with a simulation time of 80$mseg$, where $\Theta(t)$ is the step function. The results are shown in supplementary figure 9, where the dynamical variable and the computed driving force by using Eq. (23) are shown with solid lines, and the estimated values from Eq. (**36**) is shown by circles. Notice that Eq. (**36**) is evaluated with the characteristic curves which were previously estimated with the pure sinusoidal dynamical function, i.e. we only modify the dynamical variable but the Fourier coefficients and the A_0, A_1 and A_1' remain fixed. This remark can be written more explicitly as $f(\hat{i}(t); \{a_k, b_k\}_{k \in [0,40]}, A_0 = 0, A_1 = 4)$ and $g(\hat{i}(t); \{a_k, b_k\}_{k \in [0,40]}, A_0 = 0, A_1 = 4, A_1' = 400\pi)$. Supplementary figure 10 shows the error between the theoretical values and the predicted values by the model. Notice that the peaks corresponds to the discontinuity jumps of supplementary figure 9, this is in agreement with the previous remark that the greater errors come from the Gibbs phenomenon. As a final result of this test, we present the V-I curve in

supplementary figure 11. This figure shows more clearly the presence of the Gibbs phenomenon at the discontinuities. A second test is presented in the following in order to visualize the kind (ii) of simulation. We set a driving force $\hat{v}(t) = 300\sin(2\pi 50\, t) + 90$ and calculate the corresponding dynamical variable $\hat{i}$, with the initial condition $i(0) = 2.5A$. We use Eq. (**38**) of the main text to obtain the corresponding dynamical variable. The results are shown in supplementary figure 12, where the solid line corresponds to the theoretical simulation of Eq. (23) and the circles are the predicted values of the model by using Eq. (**38**). The RMSE is 0.049, where the mayor discrepancy is around the discontinuity at $i = 1A$, this is attributed to the Gibbs phenomenon mentioned above. The dataset for the simulation is obtained with a simulation step $T_s = 10\ \mu seg$ and a length $N = 4000$. The predicted values by using Eq. (**38**) is plotted every 100 μseg for ease of visualization, this means that there is 400 circles in the figure.

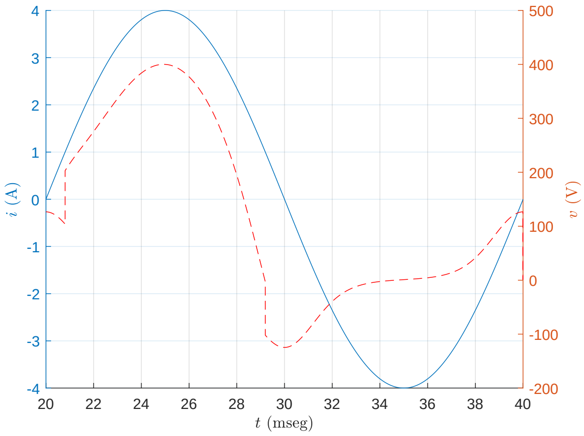

Supplementary figure 3. Temporal evolution of the discontinuous RL system. The dynamical variable $i(t)$ and the response $v(t)$ are shown with solid and dashed lines, respectively.

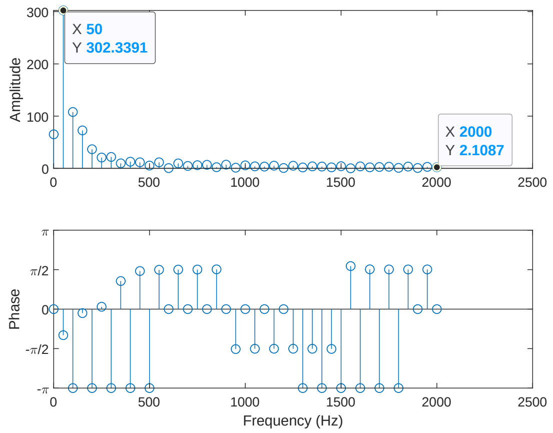

Supplementary figure 4. Single-sided amplitude and phase of the response $v(t)$ for the discontinuous RL system.

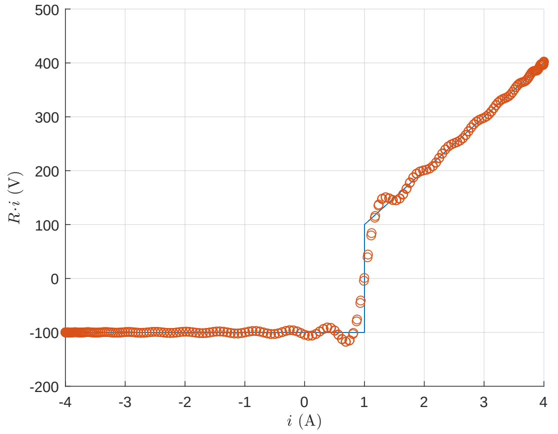

Supplementary figure 5. Characteristic curve $f(i) = R(i) \cdot i$ for the discontinuous RL system. The theoretical values of Eq. (21) and the calculation by using Eq. (**36**) of the main text are shown by solid lines and circles, respectively. RMSE $= 9.66$.

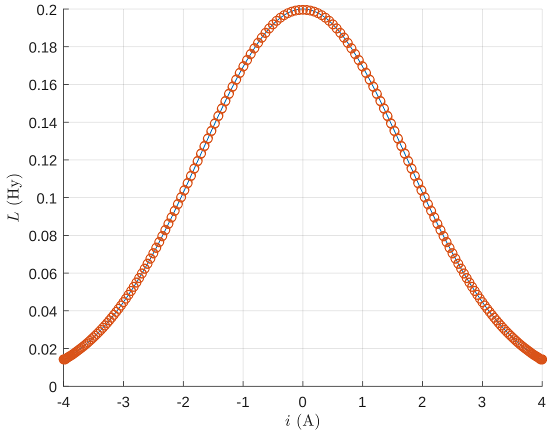

Supplementary figure 6. Characteristic curve $g(i) = L(i)$ for the discontinuous RL system. The theoretical values of Eq. (22) and the predicted values by using Eq. (**36**) of the main text are shown by solid lines and circles, respectively. RMSE $= 1 \times 10^{-5}$.

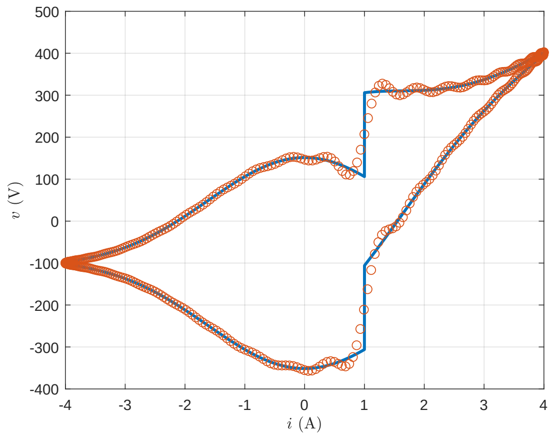

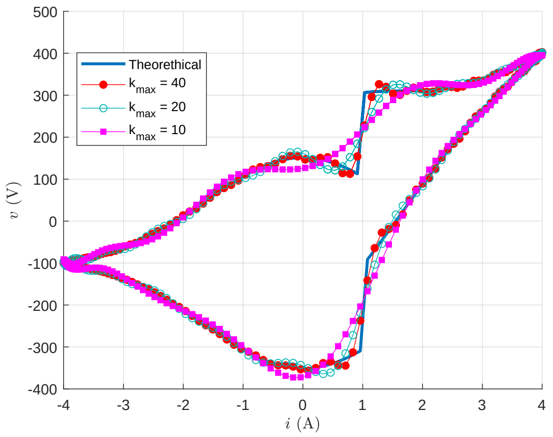

Supplementary figure 7. V-I curve for the discontinuous RL system with the sinusoidal dynamical variable. The theoretical simulation and the predicted values by using Eq. (**36**) are shown by solid lines and circles, respectively. RMSE $= 9.66$.

Supplementary figure 8. V-I curves for different numbers of maximum harmonics considered in the modeling. RMSE($k_{max} = 10$) $= 22.4$, RMSE($k_{max} = 20$) $= 15.6$ and RMSE($k_{max} = 40$) $= 9.66$.

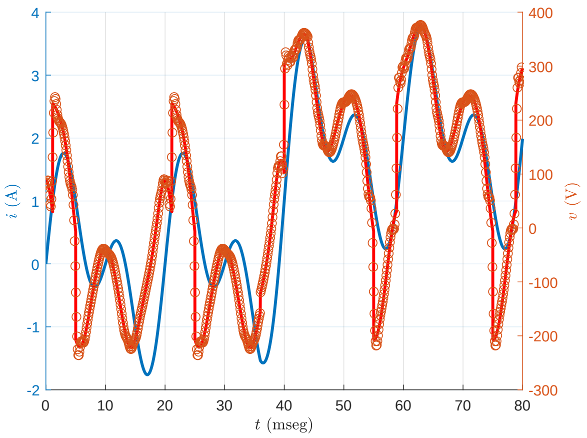

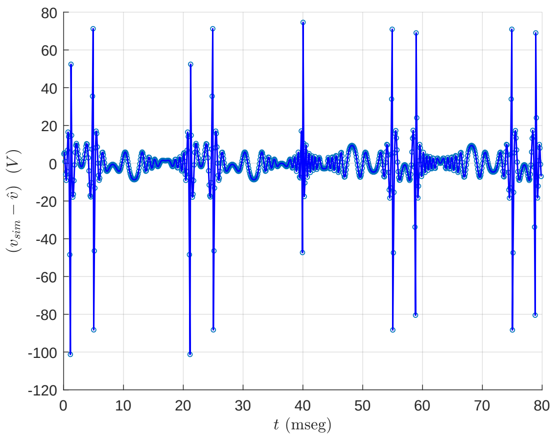

Supplementary figure 9. Time-dependence of the discontinuous RL system with a dynamical variable $\hat{\imath}(t) = \sin(2\pi 50\, t) + \sin(2\pi 100\, t) + 2\Theta(t - 2/50)$. The dynamical variable is shown by the blue solid line. The theoretical response from Eq. (23) and the simulation results by using Eq. (**36**) of the main text are shown by red solid line and circles, respectively. RMSE $= 14$.

Supplementary figure 10. Time-dependence of the error between the simulation and the predictions with the model of Eq. (**36**) of the main text for the dynamical variable $\hat{\imath}(t) = \sin(2\pi 50\, t) + \sin(2\pi 100\, t) + 2\Theta(t - 2/50)$. RMSE $= 14$.

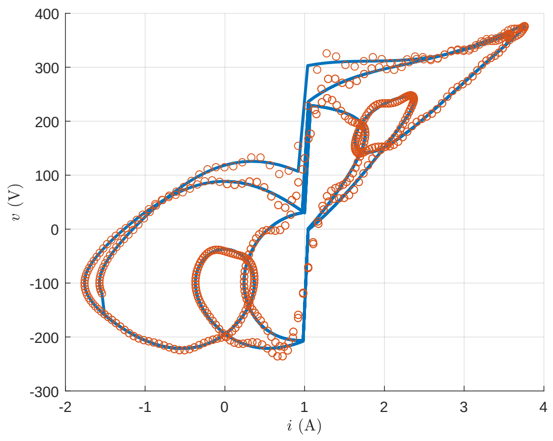

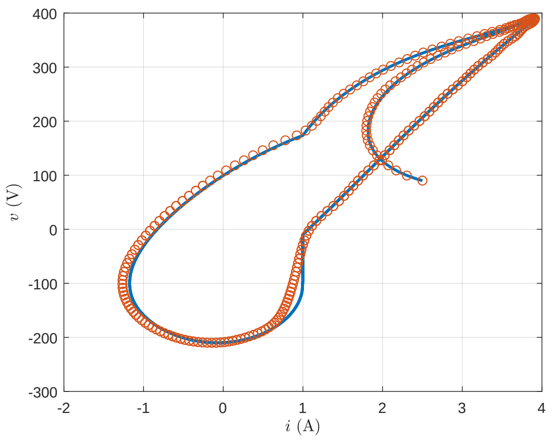

Supplementary figure 11. V-I curve for the discontinuous RL system for a dynamical variable $\hat{\imath}(t) = \sin(2\pi 50\, t) + \sin(2\pi 100\, t) + 2\Theta(t - 2/50)$. The theoretical simulation and the predicted values by using Eq. (**36**) of the main text are shown by solid lines and circles, respectively. RMSE $= 14$.

Supplementary figure 12. V-I curve for the discontinuous RL system for a driving force $\hat{v}(t) = 300 \sin(2\pi 50\, t) + 90$ and $i(0) = 2.5$ A. The theoretical simulation and the predicted values by using Eqs. (**36**) and (**38**) of the main text are shown by solid lines and circles, respectively. RMSE $= 0.049$.

Diode

An ideal diode is one of the simplest devices which can be defined by a nonlinear I-V curve. In this section, we apply the RC parallel system to model a diode (see the section *Nonlinear parallel RC system* of the main text). The nonlinear R component represents the static nonlinear I-V curve of the diode, and the C component can be associated to a loss capacitor. Consider the dynamical variable $v(t) = A_1 \sin(\omega t)$, with $A_1 = 0.8$ V and $\omega = 2\pi 50$ rad/seg. The temporal evolution of this dynamical variable and the driving force, which corresponds to the diode current, are shown in supplementary figure 13. The FFT of the driving force is shown in supplementary figure 14. It can be seen that the amplitude is negligible at 1000 Hz, which corresponds to the 20th harmonic, however we will choose up to the 40th harmonics consistent with the previous example. Now, we use Eq. (**36**) of the main text in order to calculate the characteristic curves. In this example, the g function, which corresponds to the capacitor has neglected values. For this reason, we decide to calculate the I-V curve directly, this is shown in supplementary figure 15. We obtain $\mathrm{RMSE}(i(t) - \hat{i}(t)) = 2 \cdot 10^{-5}$. We test the model with a different dynamical variable $\hat{v}(t) = 0.3\sin(2\pi 50\, t) + 0.3\sin(2\pi 100\, t) + 0.3\sin(2\pi 150\, t)$, and as a result, we obtain an error $\mathrm{RMSE}(i(t) - \hat{i}(t)) = 6 \cdot 10^{-6}$. For a chirp dynamical variable, with 0.8 V in amplitude and an increasing frequency from 0 to 500 Hz in a simulation time of 50 mseg, we obtain $\mathrm{RMSE}(i(t) - \hat{i}(t)) = 2 \cdot 10^{-5}$.

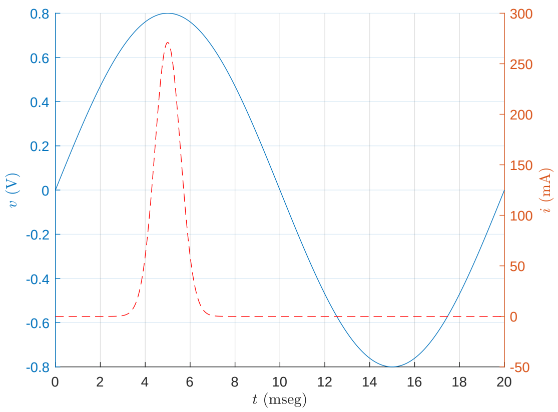

Supplementary figure 13. Time-dependence of a fundamental period of the dynamical variable $v(t)$ and driving force $i(t)$ for the circuit with a diode, with solid and dashed lines, respectively.

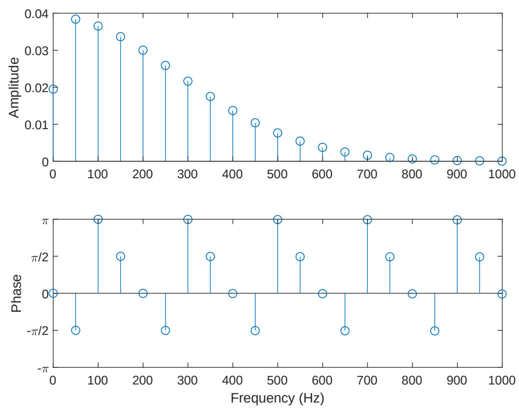

Supplementary figure 14. Single-sided amplitude and phase, in π module, of the driving force $i(t)$ for the circuit with a diode.

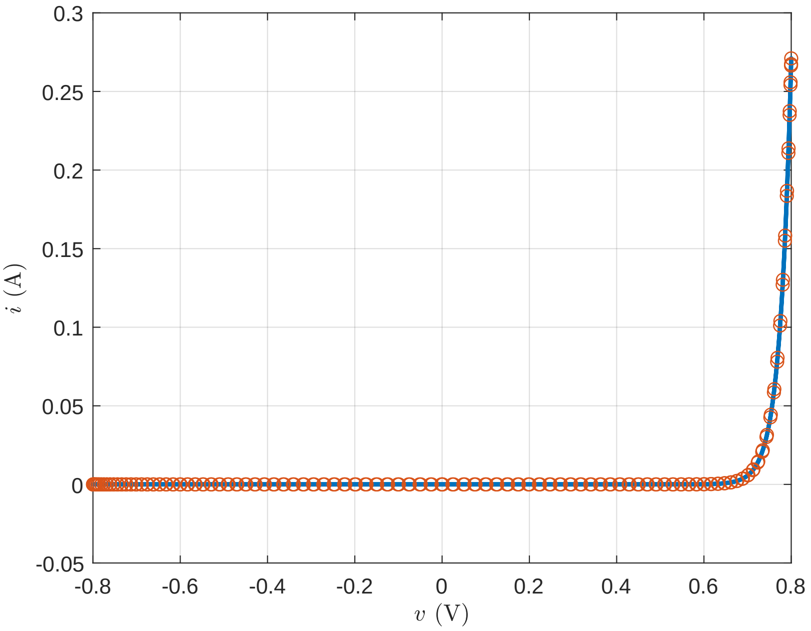

Supplementary figure 15. I-V curves for the circuit with a diode. The theoretical simulation and the predicted values by using Eq. (**36**) of the main text are shown by solid lines and circles, respectively. $\mathrm{RMSE} = 2 \cdot 10^{-5}$.

Diode in parallel with a capacitor

Consider a circuit with a diode in parallel with a 100 μF capacitor. To model the system, we apply a dynamical variable $v(t) = A_1 \sin(\omega t)$, with $A_1 = 0.8$ V and $\omega = 2\pi 50$ rad/seg. This dynamical variable and the corresponding driving force are shown in supplementary figure 16. The FFT of the driving force is shown in supplementary figure 17. Then, we use Eq. (**36**) of the main text to verify the prediction of the I-V characteristic curve, this is shown in supplementary figure 18. We obtain $\text{RMSE}(i(t) - \hat{i}(t)) = 1.1 \cdot 10^{-5}$. For a different dynamical variable, $\hat{v}(t) = 0.3\sin(2\pi 50\ t) + 0.3\sin(2\pi 100\ t) + 0.3\sin(2\pi 150\ t)$ we use the model to compute the driving force, obtaining $\text{RMSE}(i(t) - \hat{i}(t)) = 2.3 \cdot 10^{-5}$, where the correspondingly I-V curve is shown in supplementary figure 19. For a chirp dynamical variable, with 0.8 V in amplitude and an increasing frequency from 0 to 500 Hz, in a simulation time of 50 m seg, we obtain $\text{RMSE}(i(t) - \hat{i}(t)) = 2.5 \cdot 10^{-4}$, where the correspondingly I-V curve is shown in supplementary figure 20. This example shows that the estimation of the characteristic curves from the sinusoidal response allows us to obtain a model which is able to predict the response from a wide variety of new dynamical variables, in particular a chirp response. Notice that this has been possible because the system follows a first order differential equation such as that of Eq. (**44**) of the main text. However, many real systems are frequency dependent, this means that the resistive and inductive elements are explicitly frequency dependent. In these cases, the formalism of this work is expected to be valid only on a frequency range around the frequency that was used for the system modeling. See the *Discussion* section in the main text for more details.

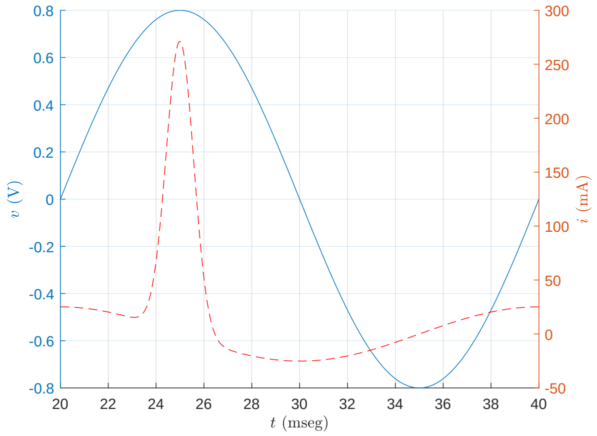

Supplementary figure 16. Time-dependence of a fundamental period for the diode in parallel with a capacitor. The dynamical variable $v(t)$ and driving force $i(t)$ are shown by solid and dashed lines, respectively.

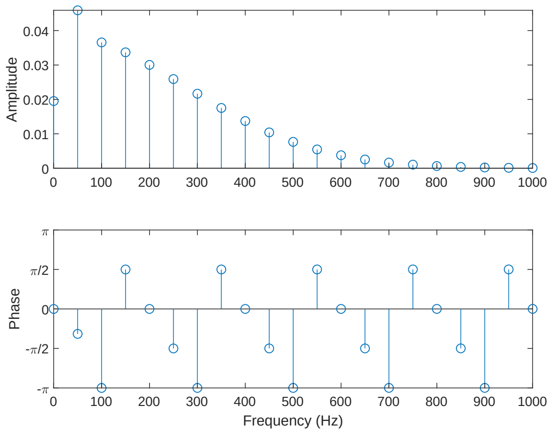

Supplementary figure 17. Single-sided amplitude and phase of the driving force $i(t)$, for the diode in parallel with the capacitor.

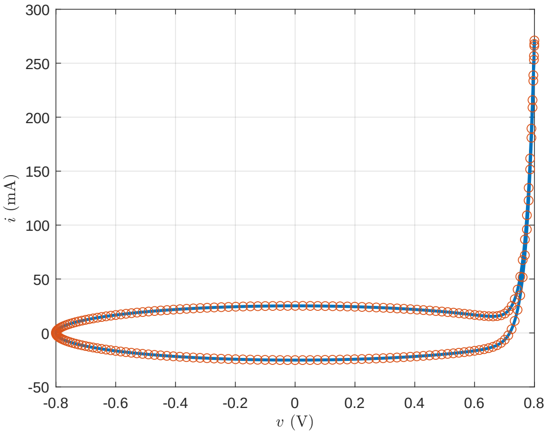

Supplementary figure 18. I-V curves for the diode in parallel with a capacitor. The theoretical simulation and the predicted values by using Eq. (**36**) of the main text are shown by solid lines and circles, respectively. $\text{RMSE} = 1.1 \cdot 10^{-5}$.

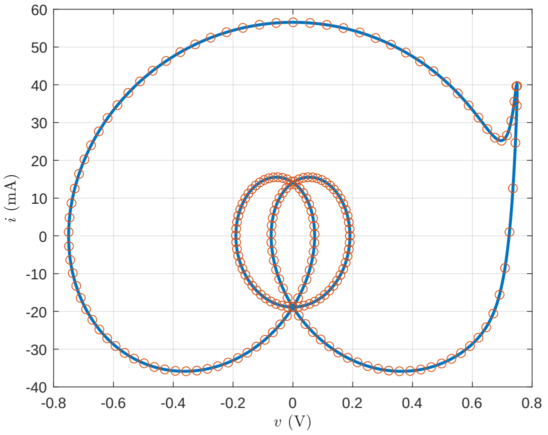

Supplementary figure 19. I-V curves for the diode in parallel with a capacitor for a dynamical variable $\hat{v}(t) = 0.3\sin(2\pi 50\ t) +$ $0.3\sin(2\pi 100 t\ ) + 0.3\sin(2\pi 150\ t)$. The theoretical simulation and the predicted values by using Eq. (**36**) of the main text are shown by solid lines and circles, respectively. $\text{RMSE} = 2.3 \cdot 10^{-5}$.

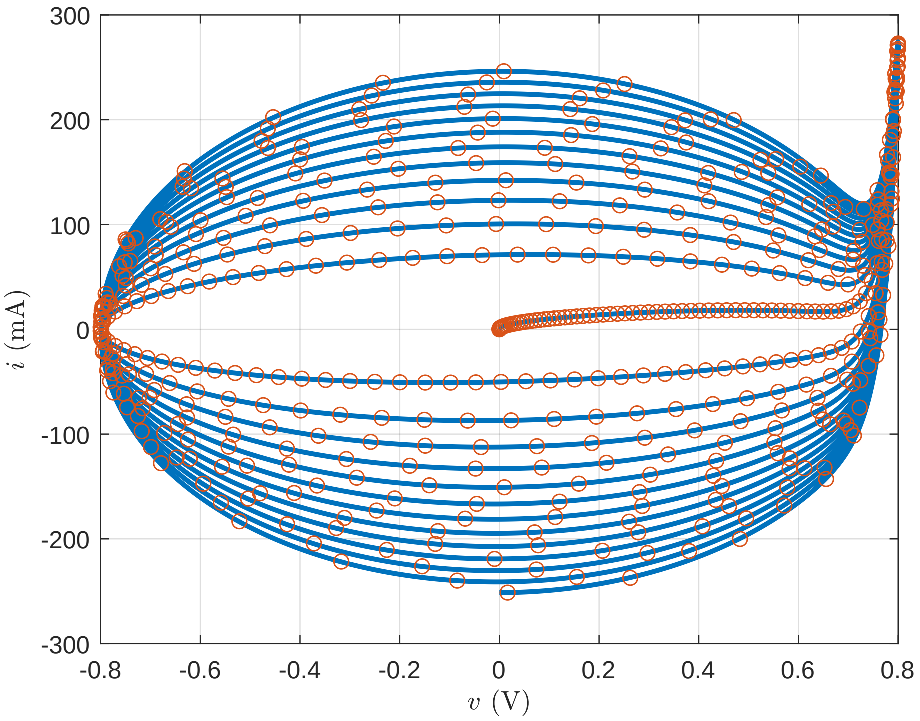

Supplementary figure 20. I-V curves for the diode in parallel with a capacitor, sourced by a chirp dynamical variable $v(t)$. The theoretical simulation and the predicted values by using Eq. (**36**) of the main text are shown by solid lines and circles, respectively. RMSE $= 2.5 \cdot 10^{-4}$.

Nonlinear inductor

In the main text, we have shown that the Fourier analysis of the driving force is equivalent to the polynomial regressions of the characteristic curves, and the polynomial regressions are known to present the Runge phenomenon. The aim of this example is to show the presence of the Runge phenomenon. This section studies a nonlinear inductor in series with a resistance of 0.1 Ω. We use the constitutive equation for the inductor

$$v_L = L(i)\, i', \tag{25}$$

where

$$L(i) = 0.02\left(1 - \tanh^2(i/5)\right) . \tag{26}$$

According to Step **(1)** in figure **1** of the main text, we use a sinusoidal dynamical variable for the system modeling, in particular, we set $i(t) = A_1 \sin(\omega t)$, with $A_1 = 25$ A and $\omega = 2\pi 50$ rad/seg. The dynamical variable and the driving force are shown in supplementary figure 21. We compute the FFT of the driving force according to Step **(2)**, this is shown in supplementary figure 22. It can be seen that the amplitude is almost negligible at 1500 Hz, which corresponds to the 30th harmonic, then, we consider a system modeling up to the 40th harmonic. We test the model with the same dynamical variable by using Eq. (**36**) of the main text, this is shown in supplementary figure 23. The obtained error is RMSE$(v(t) - \hat{v}(t)) = 0.37$. Supplementary figure 24 shows the characteristic curve $L(i)$ from Eq. (26) compared to the g function calculated by Eq. (**36**) of the main text. An oscillatory Runge phenomenon[7], which is typical of a polynomial curve fitting, can be observed at the extreme values. This oscillation can be improved by reducing the order k_{max}, but at the expense of a worst fit. Now, once the characteristic curves have been obtained, the model is ready to be tested. In this example, we use the kind (i) of simulation according to figure **1** of the main text. In the following we apply dynamical variables restricted to $|i(t)| < 20$ A in order to avoid the range of values where the Runge oscillation is present. Firstly, we consider the sum of two sinusoidal functions $\hat{i}(t) = 10\sin(\omega t) + 10\sin(2\omega t)$, where the V-I curve is shown in supplementary figure 25, and the obtained error is RMSE $= 2.37$. Secondly, we test a dynamical variable $\hat{i}(t) = 10\sin(\omega t) + 10\sin(2\omega t) - 5\Theta(t - 3/50)$, where Θ is the step function. The time-dependence of the dynamical variable and the corresponding driving force is shown in supplementary figure 26. Notice that, after three fundamental periods the step function has a non-neglected value and the function is shifted by -5 A. The result of the V-I curve is shown in supplementary figure 27, we obtain RMSE$(v(t) - \hat{v}(t)) = 2.96$. Finally, this section has presented an example that shows the presence of the Runge phenomenon at the edges of the interval $[A_0 - A_1, A_0 + A_1]$. If this oscillation is problematic, a solution may be to restrict the new dynamic variables for testing and simulation to a narrower range.

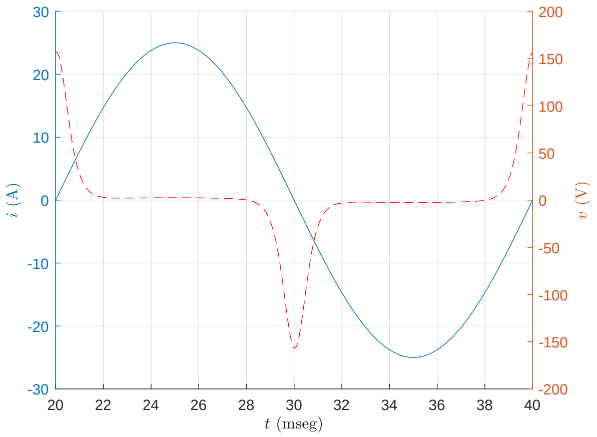

Supplementary figure 21. Time-dependence of the dynamical variable $i(t)$ and the response $v(t)$, shown with solid and dashed lines, respectively.

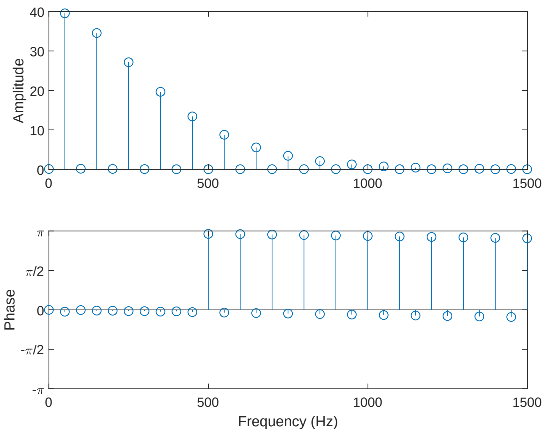

Supplementary figure 22. Single-sided amplitude and phase of the response $v(t)$ for the nonlinear inductor.

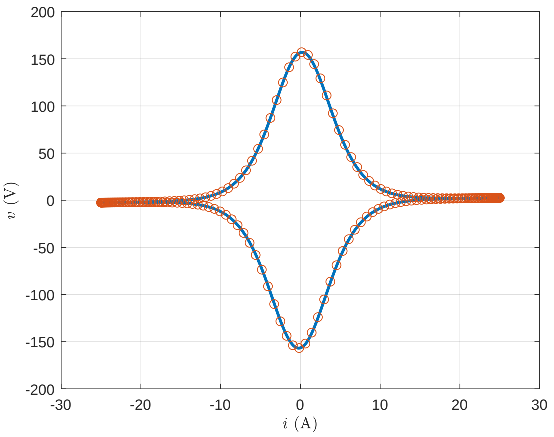

Supplementary figure 23. V-I curve for the nonlinear inductor with the sinusoidal dynamical variable $i(t) = 25\sin(\omega t)$. The solid line corresponds with simulation data and the circles are the predicted values by using Eq. (**36**) of the main text. RMSE $= 0.37$.

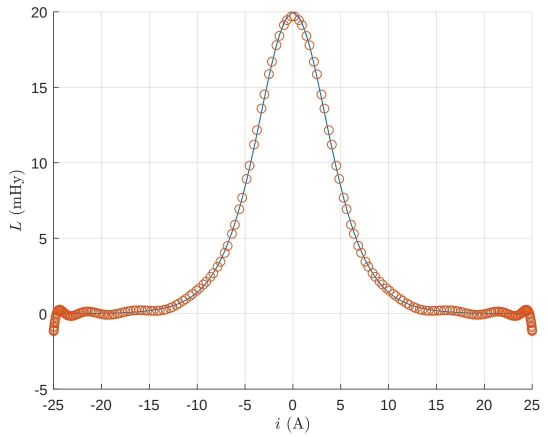

Supplementary figure 24. Characteristic curve $L(i)$ for the nonlinear inductor. The theoretical simulation by using Eq. (26) and the predicted values by using Eq. (**36**) of the main text are shown by solid lines and circles, respectively.

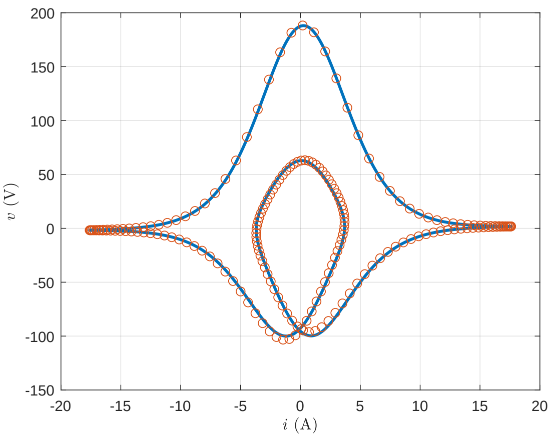

Supplementary figure 25. V-I curve for the nonlinear inductor with a dynamical variable $\hat{i}(t) = 10\sin(\omega t) + 10\sin(2\omega t)$. The theoretical simulation and the predicted values by using Eq. (**36**) of the main text are shown by solid lines and circles, respectively. RMSE $= 2.37$.

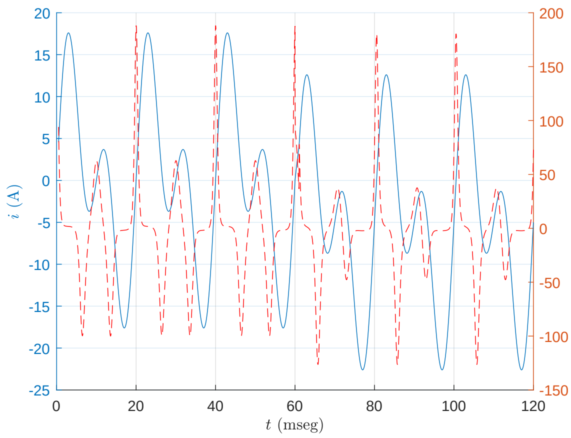

Supplementary figure 26. A dynamical variable $\hat{i}(t) = 10\sin(2\pi 50\, t) + 10\sin(2\pi 100\, t) - 5\Theta(t - 3/50)$, and the theoretical values of driving force $v(t)$ are shown by solid and dashed lines, respectively.

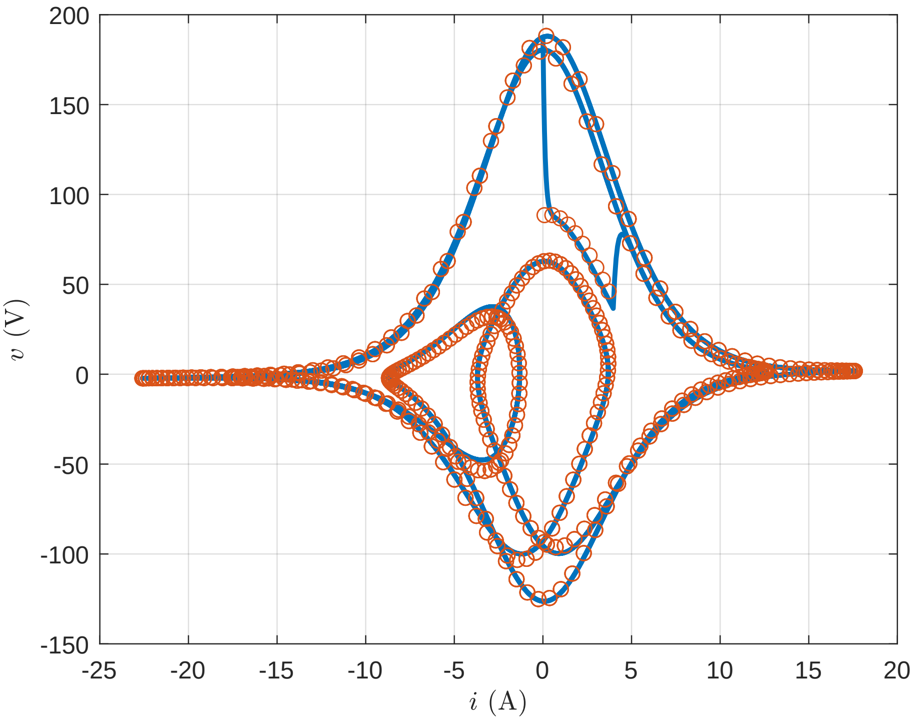

Supplementary figure 27. V-I curve for the nonlinear inductor with $\hat{i}(t) = 10\sin(\omega t) + 10\sin(2\omega t) - 5\Theta(t - 3/50)$. The theoretical simulation and the predicted values by using Eq. (**36**) of the main text are shown by solid lines and circles, respectively. RMSE $= 2.96$.

References

1. Cooley, J., Lewis, P. & Welch, P. The finite fourier transform. *IEEE Transactions on Audio Electroacoustics* **17**, 77–85, DOI: 10.1109/TAU.1969.1162036 (1969).
2. Shannon, C. Communication in the presence of noise. *Proc. IRE* **37**, 10–21, DOI: 10.1109/JRPROC.1949.232969 (1949).
3. Stoica, P. & Moses, R. *Spectral Analysis of Signals* (Prentice Hall, Upper Saddle River, NJ, 2005).
4. Gibbs, J. W. Fourier's series. *Nature* **59**, 200–200 (1898).
5. Gibbs, J. W. Fourier's series. *Nature* **59**, 606–606 (1899).
6. Hewitt, E. & Hewitt, R. E. The Gibbs-Wilbraham phenomenon: An episode in fourier analysis. *Arch. Hist. Exact Sci.* **21**, 129–160 (1979).
7. Runge, C. Über empirische funktionen und die interpolation zwischen äquidistanten ordinaten. *Zeitschrift für Math. und Physik* **46**, 224–243 (1901).
